# Biomarker discovery for tuberculosis using metabolomics

**DOI:** 10.3389/fmolb.2023.1099654

**Published:** 2023-02-20

**Authors:** Yi Yu, Xin-Xin Jiang, Ji-Cheng Li

**Affiliations:** ^1^ Center for Analyses and Measurements, College of Chemical Engineering, Zhejiang University of Technology, Hangzhou, China; ^2^ Clinical Research Laboratory, Shaoxing Seventh People’s Hospital, Shaoxing, China; ^3^ Institute of Cell Biology, Zhejiang University Medical School, Hangzhou, China

**Keywords:** tuberculosis, *Mycobacterium tuberculosis*, metabolomics, biomarker, drug resistant tuberculosis, pulmonary tuberculosis

## Abstract

Tuberculosis (TB) is the leading cause of death among infectious diseases, and the ratio of cases in which its pathogen *Mycobacterium tuberculosis* (Mtb) is drug resistant has been increasing worldwide, whereas latent tuberculosis infection (LTBI) may develop into active TB. Thus it is important to understand the mechanism of drug resistance, find new drugs, and find biomarkers for TB diagnosis. The rapid progress of metabolomics has enabled quantitative metabolite profiling of both the host and the pathogen. In this context, we provide recent progress in the application of metabolomics toward biomarker discovery for tuberculosis. In particular, we first focus on biomarkers based on blood or other body fluids for diagnosing active TB, identifying LTBI and predicting the risk of developing active TB, as well as monitoring the effectiveness of anti-TB drugs. Then we discuss the pathogen-based biomarker research for identifying drug resistant TB. While there have been many reports of potential candidate biomarkers, validations and clinical testing as well as improved bioinformatics analysis are needed to further substantiate and select key biomarkers before they can be made clinically applicable.

## Introduction

Until the COVID-19 pandemic, tuberculosis (TB) was the leading cause of death globally among infectious diseases. Its causative pathogen, the *bacillus Mycobacterium tuberculosis* (Mtb), can be easily transmitted *via* airborne aerosols, expelled from people who are sick with TB. Mtb mainly infect the lungs, causing pulmonary TB, which is characterized by pathologically necrotizing granulomatous inflammation in the lung. It can also infect other organs, causing extrapulmonary TB, including the deadlier tuberculous meningitis (TBM). According to the recent global tuberculosis report of the World Health Organization (WHO) (WHO, 2022), it is estimated that about one quarter of the human population worldwide has been infected with Mtb, which is about two billion people. A small proportion of the latent tuberculosis infection (LTBI) will develop into active TB. This proportion is much higher for people infected with HIV. Other risk factors such as diabetes, smoking and alcohol consumption, also enhance the probability for LTBI to develop into active TB. The new incidence of active TB in 2021 was about 10.6 million people. Statistics shows that Mtb can infect all age groups. About 90% of TB patients are adults, among whom there are more men than women (56.5% versus 32.5% in 2021). Without treatment, the mortality rate from TB disease is about 50%. The COVID-19 pandemic has worsened the situation and caused reduced access to TB diagnosis and treatment (WHO, 2022). This led to an increase of TB deaths from about 1.4 million in 2019 (the year before the pandemic) to 1.5 million in 2020 and 1.6 million in 2021, with a worsening trend in terms of TB deaths and incidence for 2022. According to the latest WHO report, nearly all TB cases can be cured, if diagnosed early and treated properly. Therefore, early and accurate diagnosis of TB and effective treatment are very important, in order to reduce the death rate and interrupt the transmission.

Given the huge impact of TB on public health, there have been global efforts from both the government side and the scientific communities. Since 2014, all member states of the United Nations (UN) have committed to ending the TB epidemic by 2030, which lends strong support to fundamental and clinical research on tuberculosis. There have been a great number of studies in the literature on biomarker discovery for TB, which is essential for diagnosis, treatment monitoring, risk analysis and prognosis. Biomarkers have also played an important role in studies of mechanism of action, drug resistance and new drug development. Not including other omics studies, there were tens of new metabolomics studies on TB every year in the past few years. Substantial progress has been made in diagnosis and the understanding of the pathogen-host interaction as well as the mechanism of drug resistance of Mtb. Here we will review recent metabolomics-based studies on biomarker discovery in TB in the past few years, since 2016. While TB is predominantly pulmonary, we kept TBM and Osteoarticular tuberculosis (OTB) in the coverage, while leaving out less deadly *Mycobacterium smegmatis* (M.smeg) infections and other diseases in the *mycobacterium* complex. These biomarkers may be used for diagnosis, therapy efficacy evaluation and treatment monitoring and outcome prediction.

## Overview of TB and metabolomics-based biomarker discovery

### Diagnosis, treatment and drug resistance of TB

Mtb is transmitted mainly *via* aerosols and are engulfed by alveolar macrophages in the lungs of the infected hosts. Subject to the immunological defense responses from the host, including hypoxia, acidification, nutrient starvation, and oxidative stress, Mtb has exhibited a strong adaptability to deal with these macrophage antibacterial responses. Studies reveal that Mtb can utilize multiple carbon and nitrogen sources from the host cells for its growth and replication, and Mtb’s central carbon metabolism plays a key role in its physiology and pathogenicity.

Currently, the diagnosis of pulmonary TB mainly relies on the detection of Mtb in sputum, based on sputum smear microscopy and bacteriological culture. Albeit a gold standard for TB diagnosis, this method takes a long time (3–4 weeks) before the result is available. Obviously, the sputum-based diagnosis is not applicable for extrapulmonary TB. Furthermore, many active TB patients do not present with Mtb-positive sputum, including TB patients co-infected with HIV, and TB patients with diabetes (Bacakoğlu et al., 2001), as well as children. The more recent positive GeneXpert MTB/RIF molecular test (Boehme et al., 2010) can provide sensitive detection of tuberculosis and rifampin resistance directly from untreated sputum in less than 2 h. However, this rapid test has not been made widely accessible. In 2020, it was used as the initial diagnostic test for only 33% of newly diagnosed TB cases. For extrapulmonary TB, the diagnosis often relies on invasive sample collection from tissues or biological fluids, e.g., pleural-, cerebral-, synovial-fluids. Therefore, developing rapid and accessible diagnostic tests with high sensitivity and specificity would be extremely important in the combat against TB.

Effective drug treatments of TB were first developed in the 1940s. Currently, there are four first-line drugs: isoniazid [INH], rifampicin [RIF], ethambutol [EMB], and pyrazinamide [PZA]. The treatment for drug-susceptible TB (DS-TB) recommended by the WHO is a 6-month regimen of the four drugs. However, based on available statistics, the WHO reports that about 15% of the treatments are not very successful. Globally, about 3%–4% of first-time TB and 18%–21% of recurring TB have been found to be rifampicin-resistant (RR) or multidrug-resistant (MDR, which shows resistance to both INH and RIF). The burden of drug-resistant TB (DR-TB) has increased between 2020 and 2021, with 450,000 new cases of RR-TB in 2021 (WHO, 2022). In addition, there are also INH-resistant TB, extensively drug-resistant TB (XDR-TB), and pre-XDR-TB. Pre-XDR-TB is both MDR and resistant to any fluoroquinolone, whereas XDR-TB meets the definition of pre-XDR-TB, plus resistance to at least one additional Group A drug of the second-line medicines (WHO, 2021). It is the drug resistance that has made it difficult to eradicate tuberculosis. Both RR and MDR necessitate the administration of second-line drugs, which may cause more negative side effects. It is essential to understand the mechanism of the drug resistance of Mtb, in order to effectively treat the disease and develop new or alternative drugs. Despite some promising results, conclusive answers are yet to be found. Furthermore, in the case of DR-TB, biomarkers that can timely identify drug resistance may enable proper early adjustment of the regimen and combined administration of second-line or other potent drugs.

### Metabolomics approaches to biomarker discovery

Biomarkers can play a key role in accurate diagnosis and prognosis of TB, in identifying LTBI, and in predicting their risk of developing into active TB. There may also be biomarkers for monitoring the treatment progress and evaluating the therapeutic efficacy. They can also be used in the mechanistic investigation of the drug resistance and development of new drugs. In recent years, biomarker discovery has been greatly facilitated by the advance of the modern multi-omics strategies, including metabolomics and lipidomics, based on quantitative liquid/gas chromatography and mass spectrometry technology. These omics technologies have been applied in the various biomedical studies, including studies on TB (Pitaloka et al., 2022), myocardial infarction (Liu et al., 2022), ischemic stroke (Montaner et al., 2020), depressive disorders (Sethi and Brietzke, 2015; Yu et al., 2021) and other diseases (Tounta et al., 2021).

Metabolomics is a modern technology developed based on technological advances in physics and analytical chemistry. It is capable of quantitative characterization of high-throughput light-weight metabolite molecules. NMR and mass spectrometry (MS) are the two main analytical platforms. By measuring the metabolite changes, metabolomics can be used to find characteristic differentially expressed metabolites, which can be used as biomarkers, for diagnosis, distinguishing between diseases, and monitoring the progress of medical therapy. Typical samples in metabolomics studies are biological fluids including blood, urine, sputum, cerebrospinal fluid (CSF), fecal wastes, and bacterial sources such as culture media. Lipidomics can be regarded as a branch of metabolomics, focusing on lipid metabolites. Among different omics strategies, metabolomics and lipidomics have the advantage that metabolite samples (e.g., blood, urine, and breath condensates, in the case of pulmonary TB for the latter) are relatively easier to collect, the metabolomes are relatively more stable, and thus allow a more complete understanding of cell functions, dysfunctions, and perturbations. Furthermore, essentially all diseases necessarily induce changes in metabolites, making quantitative metabolic profiling a practical way for biomarker discovery.

**FIGURE 1:**
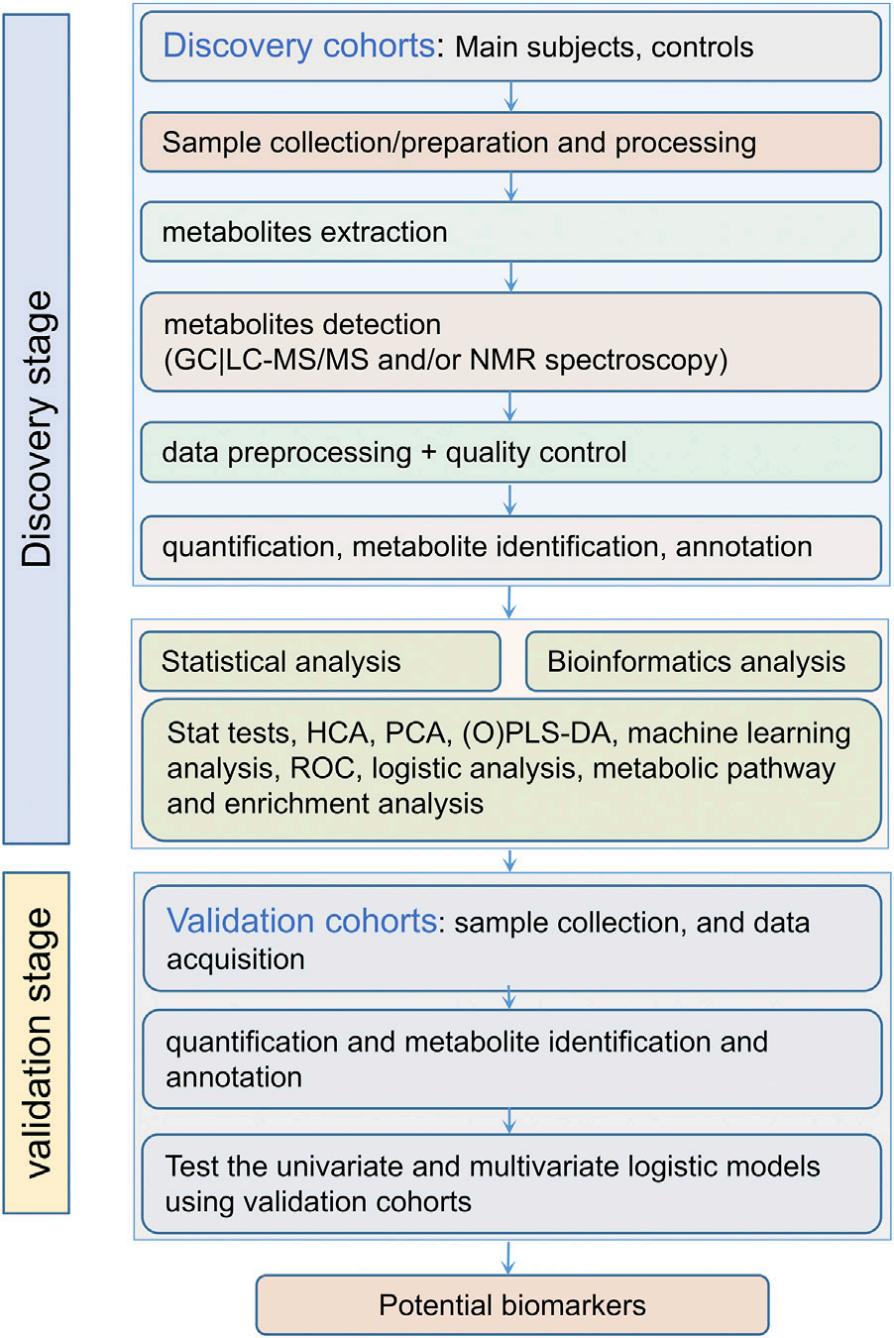
Typical work flow for biomarker discovery *via* a metabolomics approach.

The typical process of a metabolomics-based approach for biomarker discovery is shown in Figure 1. Ideally, there should be enough samples including both discovery cohorts and validation cohorts, both main research subjects (e.g., TB patients) and controls. While one can use simple screening criteria such as fold change (FC), variable importance in projection (VIP) and *p*-values to select differentially expressed metabolites, and use principal component analysis (PCA) and (orthogonal) partial least-squares discriminant analysis (PLS-DA) to analyze the difference between research subjects and controls, there are strong correlations or collinearity among these differential metabolites. Thus it is highly desirable to use more elaborate machine learning algorithms, especially those capable of eliminating redundant and uninformative variables and reducing overfitting, to select key differential metabolites as potential biomarkers. Both univariate and multivariate logistic regressions may be performed on these biomarkers. These biomarkers and the logistic models should then be tested using the independent validation cohorts. We emphasize that it is important to narrow the potential biomarkers down to a short list and have independent cross-validations.

TB research has been a highly active field, as reflected from the many reviews on this subject over the past decade. These reviews have different focuses, with each one covering part of the studies in the literature. While some covered relatively broad aspects of TB research (Goletti et al., 2016; Goletti et al., 2018; Kumar et al., 2017; du Preez et al., 2019; Kontsevaya et al., 2021), others addressed more focused topics, such as diagnostic biomarker (Haas et al., 2016; du Preez et al., 2017), treatment monitoring (Luies et al., 2017a; Pitaloka et al., 2022), drug discovery (Jansen and Rhee, 2017; Tuyiringire et al., 2018; Goff et al., 2020; Xu and Borah, 2022), mechanism of action of drugs (Awasthi and Freundlich, 2017; Yuan et al., 2021) or anti-TB compounds (Sakallioglu et al., 2021), drug resistance of Mtb and drug toxicty (Combrink et al., 2020), as well as HIV/TB co-infection (Liebenberg et al., 2021). Besides pulmonary TB, there are reviews on the status of metabolomics studies on tuberculosis meningitis as well (Zhang et al., 2018; Isaiah et al., 2020; Huynh et al., 2022).

Substantial progress has been made over the past few years, and more metabolomics studies are available, with more potential biomarker candidates discovered toward TB diagnosis, treatment monitoring and predictions, drug development, etc., which will be discussed below in this review.

## Biomarkers based on host responses

### Diagnosis of active TB

Upon infection, Mtb invades and grows inside macrophage cells. Pulmonary TB often results in pathologic lung inflammation that causes tissue damage, leading to proinflammatory and antimicrobial responses of macrophages. The metabolites of intracellular Mtb also affect macrophage functions and their response to pathogens. The role of host metabolism in regulating the inflammatory response to TB is still not well understood. Using combined metabolomics, lipidomics and cytokine profiling, it was recently found [31] that IL-1β-mediated inflammatory signaling in pulmonary TB was closely associated with remodeling of tricarboxylic acid (TCA) cycle, which was characterized by accumulation of the proinflammatory metabolite succinate and decreased concentrations of the anti-inflammatory metabolite itaconate, among other differential metabolites. This inflammatory metabolic response was particularly active in persons with MDR-TB. These findings support the concept that host metabolic remodeling is a key driver of pathologic inflammation in human TB disease.

The majority of metabolomics studies on TB are toward biomarker discovery for diagnosis of active TB. Recent studies on diagnostic biomarkers for active TB are listed chronologically in Table 1, (which included a few prior to 2016). It should be noted, however, some of these studies included LTBI as a cohort for comparison, so that they may also provide information of biomarkers for identification of LTBI. In addition, there were also studies based on the pathophysiology and the mechanism of action for the disease, and thus such studies may also be associated with drug tolerance and monitoring of the treatment progress discussed in the next section.

**TABLE 1:** Metabolomics studies on diagnostic biomarkers for active pulmonary TB.

Study	Sample/biofluids	Discovery cohorts (size n)	Metabolome fraction	Validation cohorts?	Analytical apparatus	Statistic methods	Metabolite biomarkers identified
Weiner et al. (2012)	serum	Active TB (44), LTBI (46), HC (46)	Total metabolome	Y	GC-MS or UPLC-MS/MS	*t*-test, Wilcoxon sum rank test, RF, SPLS-DA, HCA	20 metabolites including histidine, cysteine, threonine, citrulline, etc.
Che et al. (2013)	serum	TB (10), HC (10), TB before and after treatment (6)	Total metabolome	Y (TB 120, HC 120)	GC/TOF-MS	OPLS, *t*-test	5-oxoproline level lower in TB patients compared to HCs.
Frediani et al. (2014)	plasma	Pulmonary TB (17), HHC (17)	Total metabolome	N	LC-MS,/MS	*t*-test, FET, Pearson correlation, FDR, PCA, HCA, SVM	Upregulated Mtb-derived gylcolipids (trehalose-6-mycolate, phosphatidylinositol) and resolvins (RvD1, RvD2, AT-RvD1)
Zhou et al. (2015)	plasma	TB (38), T2D (40), malignancy (40), CAP (30), HC (39)	Total metabolome	N	^1^H NMR spectroscopy	PCA, OPLS-DA	26 metabolites between TB and T2D; ketone bodies, lactate, and pyruvate upregulated in TB vs. HC, but lower than in malignancy and CAP.
Sun et al. (2016)	Plasma of children	TB (28), RTI (21), HC (16)	Total metabolome	Y (TB 17, RTI 17, HC 14)	^1^H NMR spectroscopy	CART, OPLS-DA	L-valine, pyruvic acid, and betaine downregulated.
Luier and Loots (2016)	Urine	TB (46), HC (30)	MS 50–600 m/z	N	GCxGC-MS	PCA, PLS-DA, unpaired *t*-test, effect sizes	12 urinary metabolite markers, i.e., phenylacetic acid, 5-Hydroxyhexanoic acid, 2-Octenoic acid, Ribitol, 2-C-Methylglycerol, 5-Hydroxyhydantoin, Oxalic acid, L-Rhamnulose, Quinolinic acid, Indole-3-carboxylic acid, Kynurenic acid, Glycerol monostearate
Wang et al. (2017)	pleural effusion	TB (20), malignant (20), transudative (18) pleural effusions	Total metabolome	N	^1^H NMR spectroscopy	PLS-DA, OPLS-DA, one-way ANOVA, SNK and K-W test	L-Alanine, citric acid, creatine, low-density lipoprotein, aspartate, L-lactic acid, methionine, acetic acid
Che et al. (2018)	pleural effusion	TPE (51), MPE (20)	Total metabolome, semi-targeted	Y (TPE 13 + 51, MPE 13 + 8)	LC-MS/MS	PCA, OPLS-DA, *t*-test, SRC	tryptophan/kynurenine ratio
Isa et al. (2018)	urine	TB (107), asymptomatic controls (102)	Total metabolome	Y (TB 50, non-TB cough 50)	HPLC-MS	Wilcoxon rank sum test, RF, Gini importance index, RRmix, *t*-test	diacetylspermine, neopterin, sialic acid, N-acetylhexosamine
Vrieling et al. (2018)	plasma	TB (50), DM (50), TB-DM (27), HC (50)	Metabolome, targeting 225 parameters	N	^1^H NMR spectroscopy	PLS-DA, HCA, χ2 test, 1-way ANOVA, *t*-test, K-W test	ratios of histidine/phenylalanine and esterified cholesterol/sphingomyelin
Collins et al. (2018)	plasma	TB (17), HHC (16)	lipidome	N	LC-MS/MS	Wilcoxon rank sum test, FET, LIMMA, FDR	PG (16:0_18:1), Lyso-PI (18:0) and Ac1PIM1 (56:1) upregulated.
Beccaria et al. (2018)	human breath	TB (17), Mtb-negative controls (13)	metabolome	Y (TB 15, controls 5)	GC-MS, chemometric techniques	PCA, HCA, linear SVM, PLS-DA, RF	Panel of 23 breath molecules
López-Hernández et al. (2019)	Serum lipids	TB (10), TB-T2D (10), T2D (9), control (10)	Lipidome	N	UPLC-MS/MS	PCA, PLS-DA, RF, SAM, FDR, χ2 or FET, one-way ANOVA	14 glycerophospholipids, including upregulated LPC(18:1), LPC(18:0), downregulated PC(18:1/20:4), PC(18:0/18:1), PC(16:0/22:6), PC(16:1/22:6), PC(18:2/18:2), etc.
Huang et al. (2019)	Plasma	TB (35), CAP (35), LC (31), HC (35)	Total metabolome	N	UPLC-MS/MS	PCA, OPLS-DA, STT, χ2 test	Xanthine, 4-Pyridoxate, and d-Glu
Vrieling et al. (2019)	Plasma	TB (49), TB-DM (19), HC (48)	amine and acylcarnitine in plasma (targeted)	N	LC-MS/MS	PCA, HCA, FDR, 1-way ANOVA, χ2 test, PLS-DA	ratios of phenylalanine/histidine, citrulline/arginine, and kynurenine/tryptophan; citrulline, ornithine downregulated in TB and TB-DM, choline, serine, glycine, homoserine threonine lower in TB-DM than TB.
Cho et al. (2020)	Serum	TB (21), LTBI (20), HC (28)	Total metabolome	N	LC-MS/MS	PCA-DA, FDR, *t*-test, K-W or MWUT	glutamate, asparagine, sulfoxy methionine, aspartate, glutamine, methionine, ratios of glutamate/glutamine, sulfoxy methionine/methionine, and aspartate/asparagine
Luo et al. (2020a)	pleural effusion	TPE(10), LC (10),	Total metabolome, lipidome	Y (TPE 30, LC 30)	UPLC-MS/MS	PCA, FDR, PLS-DA	phenylalanine, leucine, PC(35:0), and SM(44:3)
Collins et al. (2020)	plasma	DS-TB (89), LTBI (20), HC (37), MDR-TB (85) || refugees treated for LTBI (28), HHC (69), untreated LTBI (30), HC (39)	Total Metabolome	N	MS/MS	Wilcoxon rank-sum test, Wilcoxon signed-rank test,	plasma tryptophan downregulated, kynurenine/tryptophan ratio and kynurenine upregulated.
Ding et al. (2020)	human and mouse blood, zebrafish larvae	human TB (20), mice, whole zebrafsh larvae, and respective HCs	Total Metabolome	N	MS, NMR spectroscopy	PLS-DA, *t*-test	methionine, asparagine, cysteine, threonine, serine, tryptophan, leucine, citrulline, ethanolamine and phenylalanine
Han et al. (2021)	plasma	TB (34), Lung cancer (25), CAP (30), HC (30)	lipidome	N	UPLC-MS/MS	PCA, OPLS-DA, K-mean clustering, *t*-test, FDR	PC(12:0/22:2), PC(16:0/18:2), CE (20:3), and SM(d18:0/18:1)
Luo et al. (2020b)	serum	TB (125), LTBI (101)	iron metabolism	Y (TB 66, LTBI 53)	ROCHE COBAS 8000	MWUT or χ2 test	combination of iron metabolism indexes and TBAg/PHA ratio
Albors-Vaquer et al. (2020)	serum	TB (15), HHC (30), HC (35)	Total Metabolome	N	^1^H NMR spectroscopy	PCA, OPLS-DA, *t*-test, SUS-plots	Amino acids (alanine, lysine, glutamate and glutamine), citrate, choline downregulated in TBs.
Chen et al. (2020)	Exhaled breath particles	TB (19), non-TB (17)	Total metabolome, lipidome	N	LC-MS	PCA, SAM, SVM	> 400 features selected
Izquierdo-Garcia et al. (2020)	urine	High field: TB (19), PnP (25), LTBI (17), HC (28)||	Total metabolome	Y (TB 66) ||	^1^H NMR spectroscopy (high and low-field)	PCA, PLS-DA	8 metabolites (aminoadipic acid, citrate, creatine, creatinine, glucose, mannitol, phenylalanine, hippurate)
		Low field: TB (39), PnP (31), LTBI (53), HC (29) (TB 88)		(TB 88)			
Krishnan et al. (2021)	Serum of PLWH	TB (23), controls (32)	microRNAs metabolites, cytokines/chemokines	N	UPLC-MS/MS	PCA, MWUT, FDR	gamma-glutamylthreonine and hsa-miR-215-5p
Deng et al. (2021)	urine	TB (30), LTBI (30), HC (30)	Total metabolome	Y (LTBI 16, TB 16, HC 16)	HPLC-MS	PCA, OPLS-DA, HCA, *t*-test	Glutathione and histamine
Comella-del-Barrio et al. (2021)	Urine of children	TB (6), unconfirmed TB (52), unlikely TB (4), HC (55)	Urine metabolites	N	^1^H NMR spectroscopy	PCA, PLS-DA, *t*-test, K-W tests	Differences in urine metabolic fingerprints identified
Liu et al. (2021)	pleural effusions	TB-PE (17), malignant PE (17)	Total metabolome	N	GC-MS	PCA, OPLS-DA	Stearic acid, L-cystine, and citric acid
Collins et al. (2021)	plasma	MDR-TB + HIV (64), MDR-TB (21), DS-TB (89), LTBI (20), HC (37), at different times of treatment	Total metabolome, lipidome	N	UPLC-MS/MS	Wilcoxon Rank Sum test, Wilcoxon Signed-Rank test, FDR, HCA	Increased succinate, fumarate, malate, AKG, IL-1β, LPLs, ARA, succinate/itaconate ratio, decreased itaconate, phospholipids
Jiang et al. (2021)	serum	TB (30), LTBI (30), HC (30)	Total metabolome, assisted by transcriptomics data	N	GC-MS, UPLC-MS	PCA, OPLS-DA, LASSO, χ2 test or FET, ANOVA or *t*-test, Wilcoxon signed rank test or K-W test	5-hydroxyindoleacetic acid, isoleucyl-isoleucine, heptadecanoic acid, indole acetaldehyde, 5-ethyl-2,4-dimethyloxazole, and 2-hydroxycaproic acid
Wang et al. (2022)	Intestinal flora, blood	PTB (58), HC (36)	Gut microbiome, fecal metabolome	Y (PTB 25, HC 16)	GC-MS, gene sequencing	PCoA, PCA, OPLS-DA, RF-based classification, FDR	4 genera (*Fusobacterium*, fusicatenibacter, tyzzerella and anaerotruncus), combination of five metabolites (1-tetracosanol, 3-hydroxypicolinic acid, behenic acid, pyrophosphate and tromethamine)
Chandra et al. (2022)	Mtb strains, BMDM of mice, human THP-1 macrophages, plasma, sputum	TB (40), non-TB (40)	Total metabolome	N	UPLC-MS/MS	RF, 2-tailed *t*-test, MWUT, or 1-way ANOVA models, Tukey’s *post hoc* tests, χ2 tests, or FET	Accumulation of cholestenone in sputum could be a useful biomarker.
Magdalena et al. (2022)	serum and blood cultures	TB (15), LTBI (52), NMP (20), HC (149)	Total metabolome	N	LC-MS/MS	FDR, elastic-net model	Leucine in serum, kynurenine in stimulated blood cultures
Hu et al. (2022)	plasma	SPPT (27), SNPT (37), HC (36) (90% for training)	Total metabolome	Y (10% for validation)	UPLC-MS	PCA, OPLS-DA, SVM, RF, MLP, MWUT, K-W test, 1-Way ANOVA, χ2 test	4 biomarker combinations involving, Val-Ser, 9-OxoODE, enterostatin human, EHB, MAA, DL-norvaline, EPA, His-Pro, PGA, and 1 clinical indicator (albumin).

ARA, arachidonic acid; AKG, α-ketoglutarate; ANOVA, analysis of variance; BMDM, Bone-marrow-derived macrophages, CAP = community-acquired pneumonia; CART = classification and regression tree (analysis), CE = cholesteryl ester; DM = diabetes mellitus; DS-TB = drug sensitive TB, EHB = ethyl 3-hydroxybutyrate; EPA = eicosapentaenoic acid; FDR = hochberg and benjamini false discovery rate; FET = Fisher’s exact test, HCA = hierarchical clustering analysis; HHC = asymptomatic household contacts (without active TB), K-W test = Kruskal–Wallis test, LASSO = least absolute shrinkage and selection operator; LC = lung cancer; LC-MS = liquid chromatography-mass spectrometry; LIMMA = linear model for microarray data analysis; LPC = lysophosphatidylcholine; LPL = lysophospholipid; LTBI = latent tuberculosis infection; MAA = methoxyacetic acid; MDR = mulit-drug resistant; MLP = multilayer perceptron neural network; MPE = malignant pleural effusion; MWUT = Mann Whitney U test, NMP = non-mycobacterial pneumonia; PC = phosphatidylcholine; PCA = principal component analysis, PCoA = principal coordinate analysis, PE = pleural effusions; PG = phosphatidylglycerol, PGA = L-Pyroglutamic acid, PLWH = persons living with HIV, PnP = pneumococcal pneumonia, PTB = pulmonary TB, RF = random forest; RTI = respiratory tract infection; SAM = significant analysis of microarray; SM = sphingomyelin; SNK = student-Newman-Keuls (test), SNPT = smear-negative pulmonary tuberculosis; SPPT = smear-positive pulmonary tuberculosis; SRC = spearman rank correlation, SUS-plot = shared and unique structures plot, SVM = support vector machine (analysis), SVR = support vector regression; TPE = tuberculous pleural effusion, T2D = Type-2, diabetes; VM = viral meningitis.

#### Diagnostic biomarkers to distinguish active pulmonary TB patients from non-TB controls

The simplest control study for diagnostic biomarkers would be a *two-cohort* design, which includes a cohort of active TB patients versus healthy or non-TB controls. For pulmonary TB, the dominant form of TB, an early GC-MS-based study (Che et al., 2013) found the 5-oxoproline level in serum to be consistently lower in TB patients compared to HCs, and thus could be a potential diagnostic biomarker for active TB and an indicator of pathological damage of the lung. This study used a small training dataset, with a false positive rate of 22% for the biomarker. A few studies compared the metabolome in the plasma between active pulmonary TB patients with their asymptomatic household contacts (HHC). Using a small data set (17 TB patients + 17 HHCs), Frediani et al. (2014) reported that 61 metabolites, screening by false discovery rate (FDR) q < 0.05, were upregulated in the plasma of newly diagnosed pulmonary TB patients, including glutamate, choline derivatives, Mtb-derived cell wall glycolipids and resolvins, as compared to the HHCs, and proposed Mtb-derived glycolipids (trehalose-6-mycolate, phosphatidylinositol) and resolvins (RvD1, RvD2, AT-RvD1) as potential biomarkers. Also with a small data set (17 TB + 16 HHCs), Collins et al. (2018) found that three Mtb-associated metabolites, phosphatidylglycerol (PG) (16:0_18:1), lysophosphatidylinositol (Lyso-PI) (18:0) and acylphosphatidylinositol mannoside (Ac1PIM1) (56:1), were significantly upregulated in active TB patients, and provided excellent discrimination between TB patients and HHCs with AUC = 0.97 (in ROC analysis). These potential biomarkers have to do with the pathways associated with TB disease pathogenesis or Mtb-associated lipid metabolites. This latter study used a looser screening criterion, q < 0.2, which yielded four differential metabolites. With essentially the same sample size and experimental design but different data analysis, there were no overlap of the discovered biomarkers between these two studies.

Recently, a study (Luo Y. et al., 2020) of the iron metabolism in serum between fairly large cohorts of active TB patients (n = 191) and LTBI (n = 154) found that the combination of iron metabolism indexes and TB-specific antigen/phytohemagglutinin (TBAg/PHA) ratio (>0.22) showed AUC = 0.93 with 89% sensitivity and 90% specificity in the training data set and AUC = 0.965 with 92% sensitivity and 91% specificity in the validation set, in distinguishing active TB from LTBI. There were significantly higher levels of serum ferritin and soluble transferrin receptor and significantly lower levels of serum iron, transferrin, total iron binding capacity, and unsaturated iron binding capacity in the active TB than in the LTBI group. Different from most other metabolomics studies, this work used iron metabolism indexes, and thus was likely subject to weaker statistical fluctuations, than using individual metabolite. Nevertheless, wide scatters were apparent in these indexes as well.

Pulmonary TB may lead to pleural effusion (PE), which may also be caused by non-TB malignancy, such as lung cancer, presenting a challenge in clinic diagnosis. An integrated semi-targeted metabolomics analysis revealed distinctive metabolic signatures in PE caused by TB (n = 115) and malignancy (n = 41) (Che et al., 2018). As a potential biomarker, the ratio of tryptophan/kynurenine exhibited decent performance in differentiating tuberculous PE (TPE) from malignant PE (MPE) with sensitivity of 92.7% and specificity of 86.1%, which could be further improved by the combination with adenosine deaminase. The result indicated more activation of the downstream kynurenine metabolism. A recent non-targeted metabolomics study (Liu et al., 2021) with relatively small cohorts of TPE (n = 17) and MPE (n = 17) patients found that stearic acid, L-cystine, and citric acid may be potential biomarkers for distinguishing between TBPE and MPE, with decent sensitivity and specificity. The OPLS-DA resulted in a fairly low Q^2^ = 0.30, possibly due to the small sample size.

Luo et al (2020a) explored the metabolic characteristics of large and small extracellular vesicles (EVs) from PE *via* metabolomics and lipidomics analysis, and identified in pleural large EVs a panel of four biomarker candidates, including phenylalanine, leucine, phosphatidylcholine (PC) 35:0, and sphingomyelin (SM) 44:3, which exhibited high performance in distinguishing TPE and MPE, with AUC > 0.95. The discovery set contained *only* 10 TPE and 10 Lung cancer (LC) samples, the two groups still overlapped in the PLS-DA plot, which cast doubt on the reliability of the findings. People living with HIV (PLWH) are more susceptible to Mtb infection, with disproportionately higher morbidity and mortality. A recent study (Krishnan et al., 2021) of 23 incident TB and 32 controls adopted an integrative multi-omics approach, including miRNAomics and metabolomics, to search for more sensitive serum biomarkers for TB in advanced HIV. Differentially expressed miRNA analysis revealed 11 significantly altered miRNAs. And gamma-glutamylthreonine and hsa-miR-215-5p were identified as the optimal variables to classify incident TB cases (AUC = 0.965). However, *no* differentially abundant metabolites between the TB cases and controls were found. This suggested that the metabolome was not significantly different between TB/HIV co-infected subjects and PLWH controls, which is an important finding but clearly needs further investigations using larger samples.

Possible urinary biomarkers for pulmonary TB were also investigated using metabolomics, as compared to HCs or controls with non-TB diseases, e.g., cough. In an earlier metabolomic study of 46 TB patients compared to 30 HCs (Luier and Loots, 2016), 12 urinary metabolite markers were identified, which indicated abnormal host fatty acid and amino acid metabolism induced by TB infection, particularly changes to tryptophan, phenylalanine and tyrosine. A close inspection showed that they did not overlap with the biomarkers from other studies mentioned above. The performance of these biomarkers was not tested. In a study of TB patients compared to non-TB controls with cough (Isa et al., 2018), differential metabolites related to inflammatory intermediates were detected, as a specific immune response to tuberculosis. Random forest (RF) algorithm was used to select key differential metabolites. Diacetylspermine, neopterin, sialic acid three and N-acetylhexosamine were identified as potential urine-based biomarkers for TB from two independent patient cohorts, with AUC = 0.82 in the validation set. In addition, levels of these intermediate metabolites were found to decrease after 60 days of anti-TB treatment. They were reported to show an overall sensitivity and specificity of over 95% in discriminating TB from HCs in an independent cohort of 204 participants. This study used a larger sample size than most other studies. Unfortunately, we find no overlap of biomarkers between these two urine-based studies.

Being another non-invasive approach to diagnosis, exhaled human breath contains metabolites that can be used as biological signatures for diagnosing active pulmonary TB among suspected TB patients. In a pilot study (Beccaria et al., 2018), a panel of 23 breath molecules were identified as potential biomarkers to distinguish active TB from non-TB controls, using GC-MS methodology and chemometric techniques, as well as machine learning algorithms. In another study (Chen et al., 2020), exhaled respiratory particles were collected in liquid, in order to extract lipid molecules in lung fluid samples of TB patients and controls. Over 400 features with high segregating capacity were identified using feature selection and machine learning algorithm. The cohort size, however, in both studies, was small. The former study had only 17 TB + 13 controls and 15 TB + 5 controls in the training and validation set, respectively, which could hardly cover the real-life sample variation. The repeated cross validation could not effectively increase the sample size, which may instead produce strongly biased performance (Vabalas et al., 2019). While this study was indicative the potential of finding biomarkers from exhaled breath, however, the reported biomarkers need further investigation. For the latter study, the TB and non-TB cohorts were not separated in the PCA plots. It would be much more desirable to shrink the large number of differential metabolites to a small number of biomarkers, using, e.g., the least absolute shrinkage and selection operator (LASSO) or RF algorithms.

Recently, the differences in gut microbiome and metabolic profiles in feces of untreated active pulmonary TB patients (n = 83) and HCs (n = 52) were investigated using GC-MS and V3-V4 16S rRNA gene sequencing (Wang et al., 2022) and RF-based classification models. Considerable reductions in phylogenetic alpha diversity and the production of short-chain fatty acids, dysbiosis of the intestinal flora and alterations were found in the fecal metabolomics composition of pulmonary TB patients compared with HCs; four genera had a combined diagnostic performance with AUC = 0.81, and a combination of five metabolites demonstrated fair discrimination for pulmonary TB (AUC = 0.996) and thus could serve as potential diagnostic biomarkers as well as preventive and therapeutic targets for pulmonary TB. This was the first study of the fecal metabolic profile of pulmonary TB patients.

The metabolites of TB patients include not only those of the host, but also the pathogen. Upon infection, Mtb invades and grows in macrophages, leading to changes in the metabolome under the mutual influence of the host and the pathogen. A recent global metabolic profiling of Mtb–infected macrophages identified cholestenone, which depended on the Mtb enzyme 3β-hydroxysteroid dehydrogenase, as a host/pathogen cometabolite (Chandra et al., 2022). Sputum cholestenone levels distinguished TB patients (n = 40) from non-TB controls with TB-like symptoms (n = 40) in two geographically distinct cohorts. These findings suggested that accumulation of sputum cholestenone could be a clinically useful biomarker of TB infection. Interestingly, the plasma cholestenone level showed *no* discrimination capability. Apart from the limited sample size, this study did not use a validation set.

A latest study (Hu et al., 2022) subdivided pulmonary TB into smear-positive TB (SPPT, n = 27) and smear-negative TB (SNPT, n = 37), both of which were compared with HCs (n = 36). Precise diagnosis using conventional methods is more difficult with SNPT cases. Using UPLC-MS, it combined metabolome and clinical indicators with machine learning algorithms including RF, support vector machine (SVM) and multilayer perception neural network (MLP), and found significant enrichment of fatty acid and amino acid metabolites in the plasma of TB patients, and more serious dysfunction in fatty acid and amino acid metabolisms in SPPT than in SNPT samples. Four diagnostic biomarker combinations including ten features (two lipid/lipid-like molecules and seven organic acids/derivatives, and one clinical indicator) were selected by machine learning algorithms for distinguishing SPPT, SNPT and HC with high accuracy (83%–93% for RF and 95% for MLP). We note that the validation set (10% of the samples) was very small. Compared with biomarker metabolites discussed above, one can barely find overlap with the differential metabolites involved in the four combinations.

Ding et al. (2020) investigated the common differential metabolites for diagnosis of TB in human patients, mice and zebrafish larvae, and found significantly decreased levels of most circulating small amines in all three groups of infected subjects, as compared to their respective HCs. Ten common different metabolites were found, including methionine, asparagine, cysteine, threonine, serine, tryptophan, leucine, citrulline, ethanolamine and phenylalanine, which suggested that one can use both the mice and the zebrafish models for further investigations of the mechanism of TB in human. We note that the discrimination capability of these metabolites was not tested, though there was partial overlap with the biomarkers found in Weiner et al. (2012).

#### Diagnostic biomarkers to distinguish active pulmonary TB patients from HCs and LTBI or HHCs

Quite a few studies compared the metabolomic profiles of active TB patients and HCs as well as LTBI subjects, involving complex designs of three or more cohorts. In 2012, Weiner et al. (2012) explored the metabolome of over 400 small molecules in the serum of HCs (n = 46), LTBI (n = 46), and active TB patients (n = 44), and found evidence for anti-inflammatory metabolomic changes in TB, and concluded that 20 metabolites (selected using RF), including histidine, cysteine, threonine, citrulline, tryptophan, glutamine, aspartate, and urea, were sufficient for robust discrimination of TB patients from HCs. Meanwhile, they found increased activity of indoleamine-2,3-dioxygenase-1 (IDO-1) and decreased activity of phospholipase, and increased abundance of adenosine metabolism products, as well as indicators of fibrotic lesions in active TB as compared to LTBI. No independent validation set was available, nor was ROC analysis performed to assess the performance of these metabolites. The reported 69% sensitivity at 75% specificity was not very high. In a *targeted* metabolomics study of the serum in smaller cohorts, including active TB (n = 21), LTBI (20) and HC (28), Cho et al. (2020) found higher serum levels of glutamate, sulfoxy methionine, and aspartate and lower serum levels of glutamine, methionine, and asparagine, as well as increased ratios of glutamate/glutamine, sulfoxy methionine/methionine, and aspartate/asparagine in active TB patients compared to LTBI subjects or HCs, and identified them as potential novel serum biomarkers for rapid and non-invasive pulmonary TB diagnosis. These metabolites partially overlap with those of Weiner et al. (2012). Using GC-MS and UPLC-MS, a more recent untargeted metabolomics study (Jiang et al., 2021), assisted by transcriptomics analysis, revealed a clear separation in the OPLS-DA plots for the active TB (n = 30), LTBI (n = 30) and HC (n = 30) groups, identified 33, 7 and 49 unique differential metabolites between TB and HC, between LTBI and HC and between TB and LTBI, respectively. The LASSO regression analysis selected seven of the 33 differential metabolites between TB and HC as potential diagnostic biomarkers for TB, with a combined high AUC = 0.97. Six out of these seven metabolites were identified as 5-hydroxyindoleacetic acid, isoleucyl-isoleucine, heptadecanoic acid, indole acetaldehyde, 5-ethyl-2,4-dimethyloxazole, and 2-hydroxycaproic acid, which were associated with three significantly enriched pathways (Phenylalanine, tyrosine, and tryptophan biosynthesis, Valine, leucine, and isoleucine biosynthesis, Phenylalanine metabolism). Nevertheless, the low Q^2^ = 0.135 from OPLS-DA for HC and LTBI suggested low reliability of the model. Indeed, HC and LTBI almost completely overlapped in the 2D PCA plot. We found *no* overlap between these six biomarkers and those of Cho et al. (2020) or Weiner et al. (2012).

Furthermore, the difference in the serum metabolomic profiles among active TB, asymptomatic HHC and HC groups were also investigated using ^1^H NMR spectroscopy (Albors-Vaquer et al., 2020). Here HHC can in some sense be regarded as equivalent to LTBI. Despite a big overlap in the differential metabolites between the HC-TB pair and HC-HHC pair, the serum levels of amino acids (e.g., alanine, lysine, glutamate and glutamine), citrate and choline were found to be significantly lower in TB patients compared to HHCs. Nevertheless, the serum levels alone may not provide a strong discrimination capability as there were still significant quantitative overlaps between TB and HHC. This study contained only 15 TB patients, and had no validation cohorts. Although both this study and Cho et al. (2020) reported a lower serum level of glutamine in TB compared to HHCs, they reported opposite trends for glutamate. Such a contradiction needs further investigation.

Instead of blood samples, Deng et al. (2021) performed a urinary metabolomics study of active TB patients (n = 30), LTBI (n = 30) and non-TB controls (n = 30), and found six differential metabolites, mainly related to pathways of immune regulation and urea cycle. Based on relative quantitative levels, glutathione and histamine were identified as potential biomarkers for the diagnosis of both TB and LTBI, with AUC>0.75. The LTBI and non-TB controls strongly overlapped in the PCA plots. With AUC = 0.76, the performance of glutathione in discriminating between LTBI and non-TB controls was not high. These two metabolites did not overlap with that from the above urine-based studies. Other differential metabolites exhibited irregular behavior. Deoxyribose 5-phosphate showed a high performance in discriminating LTBI from non-TB controls, yet it essentially had no ability in distinguishing between TB and either LTBI or controls.

An NMR-based *pediatric* urinary metabolomics was also investigated recently for cohorts of presumptive TB in children (n = 62, including six bacteriologically confirmed, 52 unconfirmed, and four unlikely) and age-matched HCs (n = 55) (Comella-del-Barrio et al., 2021). Differences in metabolic fingerprint in the groups with confirmed and unconfirmed TB were observed, compared to the unlikely TB and HC groups. However, the PLS-DA plots for both high-field and low-field NMR data showed partial overlap between presumptive TB and HCs. No validation set was used. The reported accuracy (≈0.69) in discriminating between presumptive TB and controls were low, with AUC = 0.65. The low Q^2^ ≈ 0.1 (<< R^2^ ≈ 0.7) cast doubt about the reliability of the PLS-DA model (Triba et al., 2015), which could be attributed to the mixed grouping.

#### Diagnostic biomarkers to distinguish active pulmonary TB patients from HCs and other diseases

Some diseases may exhibit symptoms similar to TB and thus present a challenge in diagnosis. Wang et al. (2017) did NMR-based metabolomics profiling of TB (n = 20), malignant (n = 20), and transudative (n = 18) PE, and obtained 26 differentially expressed metabolites, predominantly involved in the metabolic pathways of amino acid metabolism, glycometabolism and lipid metabolism. A group of eight different metabolites were found to be able to distinguish between the three different types of PE. It should be noted, however, that the OPLS-DA plots showed partial inter-group overlaps, with a low Q^2^ = 0.27 (<0.5) for the TB and malignant PE comparison, and no validation set was used. The predictive performance was not evaluated with ROC curves.

In 2015, Zhou et al. (2015) did an NMR-based metabolomics study of the plasma profile of 38 TB patients and 39 HCs, as well as 110 patients with other diseases, including 40 with diabetes, 40 with malignancy, and 30 with community-acquired pneumonia (CAP), in which 26 differential metabolites were found between TB and diabetes, 27 between TB and CAP, and 24 between TB and malignancy, based on OPLS-DA without a validation set. Plasma levels of ketone bodies, lactate, and pyruvate were found to be upregulated in TB compared to HC, but still lower than in CAP and malignancy. Also increased in TB were tyrosine, phenylalanine, succinate and glutamate, while glycine and formate were downregulated. We note that there was still mild overlap between TB and malignancy in the OPLS-DA plot. In 2019, Huang et al. (2019) did plasma metabolic profiling for cohorts of HCs (n = 35) and patients with TB (n = 35), CAP (n = 35), and lung cancer (LC) (n = 31), and found three differential plasma metabolites (Xanthine, 4-Pyridoxate, and D-glutamic acid) as potential biomarkers for pulmonary TB. Recently this same group (Han et al., 2021) reported decreased plasma phospholipid levels and increased cholesteryl ester (CE) levels in patients with TB. Four lipids [PC (12:0/22:2), PC (16:0/18:2), CE (20:3), and SM (d18:0/18:1)] were identified as potential biomarkers for early diagnosis of TB, with a combined high differentiating capability (AUC≥0.91). We note that there was *no* overlap between the biomarkers found in these last two studies and those discussed earlier.

Another NMR-based metabolomics study (Sun et al., 2016) aimed to identify novel plasma metabolic markers for the diagnosis of pediatric TB, using a classification and regression tree (CART) analysis approach. It included 45 TB patients and non-TB controls consisting of 30 HCs plus 38 respiratory tract infection (RTI) patients, and identified 17 metabolites that can distinguish TB from HC and RTI. Three differential metabolites, L-valine, pyruvic acid, and betaine (downregulated), were chosen as potential diagnostic biomarkers for pediatric TB, with a decent performance in an independent validation set (sensitivity 82%, specificity 84%). Interestingly, a clear separation in the OPLS-DA plot between HCs < 5 years and HCs > 5 years revealed an age dependence of the metabolite profiles.

Type 2 diabetes mellitus (DM) is a major risk factor for developing TB. A recent study (Vrieling et al., 2018) showed that concurrent TB and type 2 diabetes (T2D) resulted in a pro-atherogenic plasma lipid profile. Using NMR-based metabolomics, plasma samples were studied for HCs (n = 50) and patients with TB (n = 50), DM (n = 50) or TB-DM (n = 27). TB-DM patients displayed metabolic characteristics of both wasting and dyslipidemia. These metabolic profile changes reflect the pathology of both TB and DM. Based on PLS-DA and multiple linear regression analysis, the ratios of phenylalanine/histidine and esterified cholesterol/sphingomyelin were identified as markers for TB classification (with AUC > 0.85) regardless of DM status. Using targeted LC-MS/MS, the same research group (Vrieling et al., 2019) compared amine and acylcarnitine levels in plasma of HCs (n = 48) and patients with TB (n = 49) or TB-DM (n = 19) at the time of diagnosis and during antibiotic treatment. The ratios of phenylalanine/histidine, citrulline/arginine, and kynurenine/tryptophan discriminated TB from HC. The latter two ratios were different from their previous findings. The levels of citrulline and ornithine were found low for both TB and TB-DM, compared to HC, and the levels of choline, glycine, serine, threonine and homoserine were lower in TB-DM than in TB, and did not return to normal during antibiotic treatment. Note that in both studies, partial overlaps existed in pairwise PLS-DA plots of the metabolic profiles between groups, with no validation set. An untargeted lipidomic study (López-Hernández et al., 2019) investigated glycerophospholipid metabolism changes in patients (n = 10 out of 39 in total) with concurrent TB and DM. It was reported that TB patients shared a common glycerophospholipid profile characterized by a decrease in PCs, independent of their DM status. The observed insensitivity to DM status was consistent with Vrieling et al. (2018). Altogether, 14 glycerophospholipids, differentially deregulated in TB and TB-DM patients, could be potential biomarkers. We found no overlap between these markers than those discussed above. With only 9 or 10 subjects in each group, the sample size was small. Furthermore, there was neither independent validation cohorts nor evaluation of the performance using ROC analysis.

With an unbiased metabolomics approach, Collins et al. (2020) recently studied the metabolic profiles of plasma samples of HCs, HHCs, LTBI, and patients with drug-sensitive (DS)-TB or MDR-TB, and reported that the tryptophan pathway is highly regulated throughout the spectrum of TB infection and disease, which was characterized by increased catabolism of tryptophan to kynurenine in both active TB and LTBI, along with simultaneous increase in the expression of IDO-1. Therefore, the levels of plasma kynurenine, tryptophan, and the ratio of kynurenine/tryptophan could be a target for biomarker development as well as host-directed therapies. The analysis was simply based on the absolute metabolite level or FC and *p*-values from the Wilcoxon rank-sum test or Wilcoxon signed-rank test. The ROC curve analysis was calculated using logistic regression with 2-fold cross validation. Further analysis with OPLS-DA and other machine learning algorithms would be desirable.

Very recently (Magdalena et al., 2022), a targeted metabolomics analysis of serum and Mtb antigen-stimulated blood cultures of HCs (n = 149) and pediatric patients with active TB (n = 15), LTBI (n = 52), and non-mycobacterial pneumonia (NMP) (n = 20) found upregulation of leucine and kynurenine and downregulation of citrulline and glutamine in serum and blood cultures of TB and LTBI groups. LTBI also featured downregulation of valine in blood cultures. In contrast, the NMP metabolite profile featured an increase in citrulline and glutamine and a decrease in leucine, kynurenine and valine concentrations. Thus, using an elastic net model, leucine in serum (AUC = 0.62) and kynurenine in stimulated blood cultures (AUC = 0.72) were identified with the highest discriminatory potential for diagnosing Mtb infection (TB + LTBI vs. HC + NMP). However, we find that these AUC values were low, with very weak discriminating capability. The small TB group size did not allow the selection of the most informative metabolites for the TB versus LTBI + NMP + HC comparison.

A recent NMR-based urinary metabolomics study (Izquierdo-Garcia et al., 2020) included 40 HCs and patients of TB (n = 189), pneumococcal pneumonia (PnP, n = 42) and LTBI (n = 61). Eight differential metabolites (aminoadipic acid, citrate, creatine, creatinine, glucose, mannitol, phenylalanine, and hippurate) were identified as potential biomarkers for the diagnosis of TB, with a high capability in differentiating TB from PnP, LTBI and HC. The PLS-DA based model correctly classified 84% of the TB patients in the TB group of the validation set. However, no ROC analysis was performed to evaluate the performance of the model.

#### Diagnostic biomarkers to distinguish active extrapulmonary TB patients from HCs and other diseases

Compared to pulmonary TB, extrapulmonary TB has a much lower incidence rate. Here we focus on TB meningitis (TBM), as listed in Table 2. Using targeted GC-MS, a metabolomics study (Mason et al., 2017) of the amino acid profiles in the CSF of children infected with TBM (n = 33) as well as that of HCs (n = 34) identified five amino acids (alanine, asparagine, glycine, lysine, and proline), which were significantly elevated in TBM cases, as potential biomarkers for earlier diagnosis. Using ^1^H NMR spectroscopy, metabolomic profiling of CSF in adult with TBM (n = 18) and viral meningitis (VM) (n = 20) (Li et al., 2017) identified a total of 25 key differential metabolites as potential biomarkers that can distinguish between TBM and VM. Among them, betaine and cyclohexane were rarely reported before in TBM. Another similar study (Zhang et al., 2019) included bacterial meningitis (BM) in addition, and found that 23, 6, and 21 metabolites were able to differentiate TBM from VM, BM and meningitis-negative groups, respectively, albeit with a strong overlap between these different groups of differential metabolites. The MS-based metabolomics profiling of adult CSF by Dai et al. (2017) included further a cohort of cryptococcal meningitis (CM, n = 16), in addition to TBM (n = 50), VM (n = 17) and BM (n = 17) patients. They reported 13, 16, and nine potential biomarkers, mainly involved in the metabolism of amino acid, lipids and nucleotides, which differentiate between TBM and VM, BM, and CM, respectively. Note that Mason et al. (2017) targeted amino acid profiles in children between TBM and HCs, and was *not* comparable to the rest, untargeted studies, which had different cohorts (VM, BM, and/or CM) for each study. The differential metabolites between TBM and VM should be mostly comparable between Li et al. (2017) and Zhang et al. (2019), as both used the NMR method. They shared *only* three differential metabolites with the same trends: lower levels of glucose and L-serine and higher level of lactate in TBM, while they had opposite trends for cyclohexane, acetate, L-valine and choline. The TBM vs. VM cohorts were only marginally separated in the OPLS-DA plot in Dai et al. (2017). No common differential metabolites for TBM vs. VM were found between either Li et al. or Zhang et al. and Dai et al. Such different results may reflect the fact that the sample sizes were too small.

**TABLE 2:** Metabolomics studies on diagnostic biomarkers for extrapulmonary TB.

Study	Sample/biofluids	Discovery cohorts (size n)	Metabolome fraction	Validation cohorts?	Analytical apparatus	Statistic methods	Metabolite biomarkers identified
Mason et al. (2016)	Urine (infants, children <13 years)	TBM (12) pre- and after treatment, non-TBM (12), HC (29), middle-aged females with FMS (17)	Total metabolome in urine	Y	GC-MS	PCA, PLS-DA, MWUT, 9 logistic regression models	methylcitric, 2-ketoglutaric, quinolinic and 4-hydroxyhippuric acids
Mason et al. (2017)	CSF of children	TBM (33), controls (34)	amino acid (targeted)	N	GC-MS	PCA, PLS-DA, MWUT	5 amino acids (alanine, asparagine, glycine, lysine, and proline)
Li et al. (2017)	CSF	TBM (18), VM (20)	Total metabolome	N	^1^H NMR spectroscopy	PCA, OSC-PLS-DA, *t*-test, FET	25 key metabolites
Dai et al. (2017)	CSF (adult > 18 years)	TBM (50), VM (17), BM (17), CM (16)	Total metabolome	N	UPLC-MS	PCA, OPLS-DA, *t*-test, SVR	13, 16, and 9 biomarkers between TBM and VM, BM, and CM, respectively
Zhang et al. (2019)	CSF	TBM (31), VM (29), BM (30), HC (30)	Total metabolome	N	^1^H NMR spectroscopy	PCA, OPLS-DA, Hotelling’s T^2^ test, *t*-test	23, 6, and 21 metabolites to differentiate TBM from VM, BM, and HC, respectively
Chen et al. (2022)	serum	Osteoarticular TB (30), disease control (30), HC (30)	Total metabolome	N	LC-MS/MS	χ2 test, K-W H test, OPLS-DA,	PC[o-16:1(9Z)/18:0], PC[20:4(8Z,11Z,14Z,17Z)/18:0], PC[18:0/22:5(4Z,7Z,10Z,13Z,16Z)], SM(d18:1/20:0), SM[d18:1/18:1(11Z)]

BM = bacterial meningitis; CSF = cerebrospinal fluid; CM = cryptococcal meningitis; FMS = fibromyalgia syndrome; HC , healthy control; MWUT = Mann Whitney U test, OPLS-DA = orthoganoal PLS-DA, OSC-PLS-DA = orthogonal signal correction PLS-DA, PC = phosphatidylcholine; PCA = principal component analysis; PLS-DA = partial least-squares discriminant analysis; SM = sphingomyelin; SVR = support vector regression, TBM = TB, meningitis; VM = viral meningitis.

Instead of invasive sample collection of CSF fluid, possible metabolic biomarkers in urine for TBM have been explored as well. Mason et al. (2016) investigated the pediatric urinary metabolomics for infants and children under 13 years, including cohorts of TBM (n = 12), suspected TBM but later proved negative (n = 12) and HC (n = 29). The study identified four differential metabolites (methylcitric, 2-ketoglutaric, quinolinic and 4-hydroxyhippuric acids) as potential non-invasive diagnostic biomarkers, with strong diagnostic ability. These metabolites were different from those found in Mason et al. (2017).

OTB is another extrapulmonary tuberculosis besides TBM, mainly caused by direct infection of Mtb or secondary infection of TB in other parts. Current detection method often leads to a high misdiagnosis rate. A recent preliminary metabolomics study (Chen et al., 2022) analyzed metabolites in the serum with 30 OTB patients, 30 disease controls, and 30 HCs. Five differential metabolites, PC[o-16:1(9Z)/18:0], PC[20:4(8Z,11Z,14Z,17Z)/18:0], PC[18:0/22:5(4Z,7Z,10Z,13Z,16Z)], SM(d18:1/20:0), and SM[d18:1/18:1(11Z)], which shared many lipid metabolic signaling pathways, were identified as potential biomarkers for OTB with high diagnostic efficacy (AUC up to 90%).

### Identification of LTBI

The investigations of biomarkers for LTBI were mostly included as part of more complex studies involving the comparison with active TB and HC. Some of these are thus discussed above. A few works, as listed in Table 3, however, performed only simple control studies of asymptomatic HHCs, as compared with HCs, searching for biosignatures that can predict the progress of the TB infection status.

**TABLE 3:** Metabolomics studies on biomarkers for identification of LTBI.

Study	Sample/biofluids	Discovery cohorts (size n)	Metabolome fraction	Validation cohorts	Analytical apparatus	Statistic methods	Metabolite biomarkers identified
Weiner et al. (2018)	serum or plasma	TB-exposed progressors (66), HC (211)	Total metabolome	Y (progressors 31, HC 116)	UPLC-MS/MS, GC-MS	CERNO test, RF, GBM NNet, GLMNet	prognostic metabolic signatures, including cortisol, mannose, cotinine, glutamine, histidine, kynurenine
Duffy et al. (2019)	Plasma or serum	HHCs (4,466), (including progressors), controls	Total metabolome	N	UPLC-MS/MS, GC/MS	Wald test, χ2 test, FDR, Spearman’s test, RF	25 immunometabolomic signatures implicating cortisol, tryptophan, glutathione, and tRNA acylation networks
Weiner et al. (2020)	serum or plasma	HHCs (converters, non-converters)	Total metabolome	N	UPLC-MS/MS, GC-MS	Multiple testing	Metabolite biomarkers of inflammation, fatty acid metabolism, bile acids, pantothenate

CERNO = coincident extreme ranks in numerical observations; FDR = hochberg and benjamini false discovery rate; GBM , generalized boosted models; NNet = neural networks; GLMNet = elasticnet logistic regression; HC = healthy control; HHC = asymptomatic household contacts (without active TB), RF = random forest; ROC = receiver operating characteristics.

A predictive biomarker research (Weiner et al., 2018) investigated the metabolome of serum and plasma from HIV-negative, TB-exposed individuals in Sub-Saharan Africa. These individuals were classified as either progressors or HCs, depending on whether they progressed to TB 3–24 months post-exposure. Prognostic metabolic signatures were generated, consistent with development of subclinical disease prior to manifestation of active TB. While lack of a clear predictive biomarker, this work suggested that metabolic changes associated with pre-symptomatic disease may be observed as early as 12 months prior to TB diagnosis. With six differential metabolites, the model showed a predictive power with AUC = 0.73–0.92 in discriminating TB from other respiratory diseases in proximate samples (<5 months to TB diagnosis) in the validation data set. By integrating blood transcriptional profiling with serum metabolomic profiling for large cohorts of HHCs (n = 4,466) and non-human primates (NHPs), it was reported (Duffy et al., 2019) that the combined application of pre-existing transcriptome- and metabolome-based signatures more accurately predicted TB progression in the HHC cohorts and disease severity in the NHPs, and “further identified novel immunometabolomic signatures associated with TB progression in HHCs and NHPs”. Recently, Weiner et al. (2020) investigated the changes in transcript, metabolite, and antibody reactivity due to the early immune response of HHCs to Mtb infection, by combining metabolic profiling with ribonucleic acid sequencing and Mtb proteome arrays. The HHCs were divided into converter and non-converter groups, depending on whether their Mtb infection status converted later from negative to positive. Differences in metabolite profiles were identified, including changes in biomarkers of inflammation, fatty acid metabolism, and bile acids, between converters and non-converters. Pantothenate (vitamin B5) was significantly increased in tuberculin skin test (TST) non-converters compared to converters at baseline.

It should be noted, however, while fairly large cohorts were used, no *strong* metabolomics biomarkers were reported in these three studies.

### Monitoring therapeutic efficacy, treatment progression and prognosis

Biomarkers may be used to evaluate the efficacy of a therapy, monitor the treatment progression, and predict the treatment outcome, which aid in proper timely adjustment of the therapy as needed, and help to avoid premature release of patients. In this subsection, metabolites derived from anti-TB medicines will usually be present in the metabolomics data. Studies in this category are listed in Table 4.

**TABLE 4:** Metabolomics studies on biomarkers for monitoring therapy efficacy and progress.

Study	Sample/biofluids	Discovery cohorts (size n)	Metabolome fraction	Validation cohorts	Analytical apparatus	Statistic methods	Metabolite biomarkers identified
Das et al. (2016)	urine	TB (20) (2h, 6h, 12h, 24h, 36h, 48h postdose)	Total metabolome	N	GC-MS	PCA	2-aminobutyric acid identified as novel drug metabolite of EMB.
Luies et al. (2017c)	urine	TB with successful (21) and unsuccessful (10) treatment	Total metabolome	N	GCxGC-MS	MWUT, fold change, effect size	3,5-dihydroxybenzoic acid, 3-(4-hydroxy-3-methoxyphenyl) propionic acid
Luies et al. (2017b)	urine	successful (26) and unsuccessful (15) treatment	Total metabolome	N	GCxGC-MS	PCA, PLS-DA, MWUT, fold change	50 differential urinary metabolites identified at time of diagnosis (only 6 could pass joint screening)
van Laarhoven et al. (2018)	CSF, serum	TBM survivors (15), TBM non-survivors (17), controls (22)	Total metabolome	Y (Tryptophan: TBM 101, genetic: TBM 285)	LC-MS	Spearman’s test, Cox regression, PCA	CSF tryptophan higher in TBM than in controls but lowest in survivors; glucose, leukotriene B4
Wood et al. (2018)	plasma	TB (30) HC (30)	UPLC-MS	N	High resolution MS	two-tailed t-test	LPCs (16:0, 18:0) downregulated, PCs (34:1, 34:2, 34:4, 36:3, 36:4, 38:4), PGs (34:0, 34:1) upregulated.
Fitzgerald et al. (2019)	urine	TB (10), HHC (TST− 14, TST+ 12) | TB (recurrence 12, cure 15, failure 8), HC (14)	Targeted metabolome	N	LC-MS/MS	2-tailed *t*-test, one-way ANOVA with Tukey’s multiple comparison test	SLC1G abundance decreased much faster by week 1 for successful treatments compared to failed ones.
Yi et al. (2019)	Plasma	PTB (untreated 35, two-month treatment 31, cured 29), HC (35)	Total lipidome	N	UPLC-MS/MS	PCA, OPLS-DA, one-way ANOVA, χ2 test	L-histidine, arachidonic acid, biliverdin, L-cysteine-glutathione disulfide
Combrink et al. (2019)	urine	TB (23, before and after 1, 2, 4 weeks treatment)	Total metabolome	N	GCxGC-MS	PCA, ANOVA, RM-ANOVA, ASCA	39 metabolites identified *via* MS, altered stress levels, several enzymes, urea cycle, insulin level
Dutta et al. (2020)	plasma	TB (n = 16, baseline, month 1, 6), HHCs (n = 32)	Total metabolome	N	UPLC-MS/MS	RF, *t*-tests, ANOVA, SRC, multi-omics factor analysis	N-acetylneuraminate, quinolinate, pyridoxate; 4 metabolite panel (γ-glutamylalanine, γ-glutamylglycine, glutamine, and pyridoxate)
Chen et al. (2021)	plasma	TB (30, untreated, 2, 6 months treatment), HC (30)	lipidome	N	UPLC-MS/MS	OPLS-DA, MWUT, χ2 test, K-mean clustering	LPA (0:0/16:0), LPA (0:0/18:0)
Opperman et al. (2021)	urine	TB (40, untreated, 1,2, 4 weeks treatment) (28 cured, 12 failed)	acylcarnitine, amino acids (targeted)	N	GC-MS	FDR, unfolded PCA, ANOVA, *t* tests, ASCA	histidine, isoleucine, leucine, methionine, valine, proline, tyrosine, alanine, serine, and γ-aminobutyric acid
Parihar et al. (2022)	CSF	TBM (36, definite or probable; severity grade I, II, III, and outcome at 3 months), HC (18)	Total metabolome	N	^1^H NMR	PCA, χ2 test, MWUT, PLS-DA	lactate, glutamate, alanine, arginine, 2-hydroxyisobutyrate, formate, and cis-aconitate were upregulated, glucose, fructose, glutamine, and myo-inositol downregulated.
Jiang et al. (2022)	Plasma	standard therapy (15, controls), plus *Lactobacillus* casei (16 low-dose, 16 high-dose)	inflammatory cytokines and metabolomics	N	UPLC-MS/MS	χ2 test, K-W test, ANOVA, Spearman’s test, FDR, OPLS-DA	pyridoxamine, L-saccharopine, PS (19:0/22:6), MaR1, PC(16:0/20:4), PC(16:0/18:1), PC(16:0/16:0) upregulated. Phenylalanine, Nacetylmethionine, PE(16:0/20:1), and L-tryptophan downregulated in high-dose group.
Biadglegne et al. (2022)	plasma	HC (9), PTB (13), TBL (13), and Rx (6)	Total lipidome, proteins	N	TEM, SEC, HPTLC, MS/MS	One-way ANOVA	37 Mtb-originating proteins, sphingomyelins and sphingomyelins,
Brandenburg et al. (2022)	MTBC Strains, blood	PTB (23), HC (39)	TSA-containing PIs of PBMC (targeted)	N	LC-MS/MS	TBnet, MWUT, Wilcoxon matched-pair signed rank test, One-way ANOVA	TSA-containing PIs, PI 35:0 (PI 16:0_19:0 (TSA))
Meng et al. (2022)	Fecal samples	HC (49), LTBI (30), TB (41), TB (28, 2mon HRZE), TB (20, 2mon HRZE + 4mon HR)	gut microbiome and fecal metabolome	N	LC-MS/MS	*t*-test, PCA,PLS-DA, PCoA, Hypergeometric distribution test	gut *Clostridium*, *Bacteroides* and *Prevotella* and fecal *Trans*-4-Hydroxy-L-proline and Genistein increased during chemotherapy.
Shivakoti et al. (2022)	plasma	PTB (failure 46, cure 146) (75% of the samples)	Total lipidome	Y (test sets, 25% of the samples)	LC-MS	Adjusted least-square regression, FDR, RF	Two CE lipids: CE (16:0) and CE (18:2), as prognostic markers.
Diboun et al. (2022)	serum	SARS-CoV-2 infected (Post-TB 23, non-TB 132)	Total lipidome	N	LC-MS/MS	FDR, PCA, OPLS-DA	betaine and BCAAs for post-TB, serum alanine as prognostic biomarkers.

ANOVA = analysis of variance, ASCA = ANOVA simultaneous component analysis, BCAA = branched chains amino acids, CE = cholesteryl ester, CSF = cerebrospinal fluid, EMB = ethambutol, FDR = hochberg and benjamini false discovery rate, HHC = asymptomatic household contacts (without active TB), HPTLC = high-performance thin-layer chromatography, HR = isoniazid + rifampin, HRZE = all four first-line drugs, K-W test = Kruskal–Wallis test, LPA = lysophosphatidic acid, LPC = lysophosphatidylcholine, LTBI = latent TB infection, MaR1 = maresin 1, MTBC = mtb complex, MWUT = Mann Whitney U test, OPLS-DA = orthogonal PLS-DA, PBMC = peripheral blood mononuclear cells, PC = phosphatidylcholine, PCA = principal component analysis, PCoA = principal coordinate analysis, PE = phosphatidylethanolamine, PI = phosphatidylinositols, PLS-DA = partial least-squares discriminant analysis, PS = phosphatidylserine, PTB = Pulmonary TB, RF = random forest, RM-ANOVA = repeated-measures ANOVA, Rx = pulmonary TB patients after anti-TB treatment, SARS-CoV-2 = severe acute respiratory syndrome coronavirus 2, SEC = size exclusion chromatography, SLC1G = seryl-leucine core 1 *O*-glycosylated peptide, SRC = spearman rank correlation, TBL = TB lymphadenitis, TBM = TB meningitis, TEM = transmission electron microscope, TSA = tuberculostearic acid, TST = tuberculin skin test.

#### Biomarkers from urine samples

Urine is essentially the most non-invasive sample for TB therapy efficacy evaluation and treatment monitoring. Through urine metabolomics, Das et al. (2016) identified 2-aminobutyric acid (AABA) as a novel metabolite of EMB, one of the four first-line drugs, in urine samples collected at different times from a cohort of 20 newly diagnosed TB patients after receiving the drugs. In addition, they found that about 75% of these patients were found to be slow acetylators of INH. Based simply on FC, *p*-value, and effect size screening plus ROC analysis, Luies et al. (2017c) identified 3,5-dihydroxybenzoic acid and 3-(4-hydroxy-3-methoxyphenyl) propionic acid, related to gut microbiota imbalance, as two possible predictors of the treatment outcome for TB, using urine metabolomics on a small cohort of 31 drug-susceptible TB patients, with successful (n = 21) and unsuccessful (n = 10) treatment outcome (using first-line drugs). This same group (Luies et al., 2017b) performed another study with enlarged cohorts (26 successful vs. 15 unsuccessful), with urine samples taken at different times after the beginning of treatment; 50 urinary metabolite markers could be annotated. The treatment failure group featured an imbalanced gut microbiome, higher levels of metabolites associated with abnormalities in the long-chain fatty acid β-oxidation pathway, reduced L-carnitine and short-chain fatty acids, altered amino acid metabolism, and increased interferon gamma. We find that only six of the 50 metabolites could pass the combined *p*-value, VIP and FC screening. One of the two biomarkers found in (Luies et al., 2017c) was among the 50 metabolites, but could not pass the FC and *p*-value tests. The best discriminating metabolites were only vaguely discussed (including many that failed the combined screening), without assessment of their discriminating abilities. Using urine metabolomics, Fitzgerald et al. (2019) reported that a seryl-leucine core 1 *O*-glycosylated peptide (SLC1G) showed a significant abundance increase in TB patients compared to HHCs and HCs. In addition, the SLC1G levels by week one decreased much faster for successful treatment compared to failed treatment, so that SLC1G was proposed as a potential biomarker for TB treatment response. Its discriminating performance was not assessed. Further validation with larger cohorts are needed.

In a pharmacometabolomics study (Combrink et al., 2019), time-dependent drug-induced host-metabolome variations in urinary metabolome were observed in a cohort of 23 TB patients before and after 1, 2, 4 weeks intensive phase tuberculosis therapy, including reduction in the oxidative stress levels (aconitase, formylglycine-generating enzyme, α-ketoglutarate dehydrogenase, and succinate-semialdehyde dehydrogenase), upregulated urea cycle, and altered insulin production, as well as time-dependent induction and inhibition of several enzymes in response to the drugs. Altogether, 39 metabolite biomarkers were identified, which may be applied toward treatment monitoring. No further selection from these metabolites was done. Through a targeted metabolomics approach (Opperman et al., 2021), urinary acylcarnitine and amino acid profiles were analyzed for TB patients with a cured and failed treatment outcome, and at different times of the treatment process, including pre-treatment diagnosis. A group of significant differential metabolites were identified, including histidine, isoleucine, leucine, methionine, valine, proline, tyrosine, alanine, serine, and γ-aminobutyric acid. The time-dependent fluctuations of most metabolites, related to vitamin B6 deficiency and other alterations (Combrink et al., 2019), were found to exhibit a delayed onset or shift of the pattern in the successfully treated group. In comparison, the earlier onset in the failed patients was proposed to be related to genotypic and phenotypic variations in drug metabolizing enzymes, which led to poor treatment efficiency. We find that the levels of the majority of these metabolites did not evolve monotonically with time. Further systematic studies with larger cohorts are needed.

#### Biomarkers from CSF samples for TBM

A comparison (van Laarhoven et al., 2018) of metabolomes of both CSF and serum of TBM patients (n = 32) with controls (n = 22) showed that the levels of the majority of CSF metabolites were higher in TBM than in controls, especially in those who died during follow-up, and only five serum metabolites differed between TBM and controls. In contrast, CSF tryptophan concentrations were the lowest in patients who survived, compared with patients who died and with controls. The association of low CSF tryptophan with patient survival was validated using a much larger cohort (n = 101). The unusual pattern of tryptophan compared to other CSF metabolites suggested that cerebral tryptophan metabolism is important for the outcome of TBM, and can be a therapeutic target.

Lately, an NMR-based CSF metabolomics study (Parihar et al., 2022) for cohorts of 36 TBM patients and 18 HCs found 11 differential metabolites that could distinguish TBM from HC, among which 7 (lactate, glutamate, alanine, arginine, 2-hydroxyisobutyrate, formate, and cis-aconitate) were upregulated, and 4 (glucose, fructose, glutamine, myo-inositol) downregulated in the TBM, compared to HCs. These differential metabolites were able to classify the 3 months treatment result with good sensitivity and specificity (AUC = 0.99). Meanwhile, the lactate concentration in CSF was found to be correlated with clinical indices and MRI findings, including hemoglobin, CSF glucose, and infarction. We note that this study found a much smaller number of differential metabolites than van Laarhoven et al. (2018) but identified a small number of biomarkers that exhibited good discriminating ability.

#### Biomarkers from blood samples

Through a controlled plasma lipidomics study of 30 TB patients and 30 HCs, Wood et al. (2018) found decreased circulating levels of lysophosphatidylcholines (LPCs) and increased levels of PCs and PGs in the plasma of TB patients compared to HCs, suggesting an altered glycerophosphocholine remodeling involving deacylation–reacylation reactions at sn-2 of the glycerol backbone. It was proposed that these structural lipids with altered metabolism may be potential biomarkers for monitoring treatment efficacy. We note that the FCs were not far from 1.0, and only *t*-test was used to select differential metabolites. Lately, Jiang et al. (2022) found that *Lactobacillus casei* supplementation during the intensive phase of TB treatment significantly modulated inflammatory cytokines and metabolites; it lowered the concentrations of tumor necrosis factor-α, interleukin (IL)-6, IL-10, and IL-12. Plasma levels of phosphatidylserine (PS), maresin 1, PC, L-saccharopine, and pyridoxamine were significantly upregulated, while N-acetylmethionine, L-tryptophan, phosphatidylethanolamine, and phenylalanine were downregulated in the high-dose group, compared to the low-dose group and controls. The discriminating capability of these metabolites were not assessed, and there was no validation data set.

In a recent study using UPLC-MS based lipid profiling, Yi et al. (2019) identified four differential metabolites (L-histidine, arachidonic acid, biliverdin, and L-cysteine-glutathione disulfide) in plasma as potential biomarkers for cured pulmonary TB, which is important in avoiding premature discharge of a pulmonary TB patient. These lipid metabolites also exhibited time sensitivity during treatment, and thus can be applied toward treatment monitoring and efficacy evaluation. Recently, this group (Chen et al., 2021) identified plasma lipid metabolites, lysophosphatidic acid (LPA) (0:0/16:0) and LPA (0:0/18:0), as potential novel biomarkers for therapeutic efficacy evaluation of TB treatment (as well as early diagnosis of pulmonary TB), with 100% of both sensitivity and specificity (and AUC = 1.0). Comparing the treated with untreated TB patients, the discriminating performance of these markers increased with increasing treatment time.

By integrating metabolomics and transcriptomics, Dutta et al. (2020) explored the plasma metabolite profiles of children with active drug-susceptible TB (n = 16) and age- and sex-matched uninfected HHCs (n = 32), and identified three metabolites (N-acetylneuraminate, quinolinate, pyridoxate) that could distinguish TB status at different times during treatment. In addition, a set of four metabolites (γ-glutamylalanine, γ-glutamylglycine, glutamine, and pyridoxate) were identified as treatment response biomarkers, distinguishing post-treatment from pre-treatment samples with AUC = 0.86. There metabolites were found to be associated with immunoregulatory interactions between lymphoid and non-lymphoid cells, and p53-regulated metabolic genes and mitochondrial translation. Apparently, the sample size (n = 16) for the TB was somewhat too small.

A lipidomics-based study (Brandenburg et al., 2022) identified tuberculostearic acid (TSA)-containing phosphatidylinositols (PIs) as biomarkers for infection with clinically relevant Mtb complex (MTBC) strains. These marker lipids were found to have higher levels in peripheral blood mononuclear cells of TB patients compared to HCs, and decline to normal levels after successful treatment. This suggested that the levels of TSA-containing PIs can be used as a correlate for the mycobacterial burden and may potentially provide a clinically relevant tool for monitoring TB severity. PI 16:0_19:0 (TSA) differentiated pre-treatment TB from HCs with AUC = 0.78, sensitivity 70% and specificity 79%. Its abundance levels decreased significantly after the WHO-defined therapy completed or after being cured. Similar changes were observed for FA 19:0-containing PIs in PBMCs. Using unbiased LC-MS based lipidomics, host lipids in plasma of pulmonary TB patients were investigated and compared between treatment failure (n = 46) and success (n = 146, controls) groups (Shivakoti et al., 2022). It was found that treatment failure was associated with lower baseline levels of CEs and oxylipin and higher baseline levels of ceramides and triglycerides, compared to controls. Using RF algorithm, CE (16:0) and CE (18:2) were identified as potential prognostic biomarkers for prediction of TB treatment failure; they exhibited the best classification accuracy between cases and controls, with moderate AUC = 0.70 and 0.79 in the training and test sets, respectively.

The influence of pre-existing pulmonary TB case on the outcome of infection with SARS-CoV-2 were recently investigated using serum metabolomics analysis (Diboun et al., 2022). The metabolomic profiles of 23 COVID-19 patients with existing diagnosis of TB were compared with those without TB (n = 132). The vast majority (∼92%) of post-TB individuals showed severe SARS-CoV-2 infection, with a significantly high mortality rate (52%). Betaine and branched chains amino acids (BCAAs) were identified as potential prognostic metabolic biomarkers of severity and mortality, respectively, in COVID-19 patients with existing TB. We note, however, the two groups could not be clearly separated in OPLS-DA plots, suggesting that part of the severity and mortality in the post-TB group was not related to TB.

#### Biomarkers from other samples

A recent study (Biadglegne et al., 2022) using lipidomics and proteomics approaches showed that the protein and lipid content of circulating exosomes in Mtb-infected patients exhibited TB disease and treatment status specific molecular features, suggesting the possibility of utilizing exosomes in TB diagnostics and treatment monitoring. Exosomes are mostly composed of SMs, PCs, PIs, free fatty acids, triacylglycerols, and CEs. Their relative proportions vary with the disease or treatment state. The treatment of pulmonary TB patients influenced the overall chain lengths as well as double-bond content of these metabolites. No specific metabolites had been identified as potential biomarkers yet.

Using 16S rRNA sequencing and an untargeted LC-MS-based metabolomics approach, Meng et al. (2022) analyzed the changes of gut microbiome and the alteration in fecal metabolome of active TB patients without and with treatment of different length of time, as well as LTBI and HC cohorts. *Clostridium*, *Bacteroides* and *Prevotella* were identified as biomarkers associated with fecal metabolites 4-hydroxy-L-proline and genistein in active pulmonary TB patients during the therapy with first-line drugs. The diversity of intestinal flora and their taxonomic composition changed in response to the chemotherapy, and Mtb infection dynamically regulated fecal metabolism in active TB patients during anti-TB chemotherapy. Thus, the correlation between gut microbiome and anti-TB chemotherapy may provide potential biomarkers for evaluating the therapeutic efficacy. These findings had not been tested with validation cohorts.

Note that the treatment progress or efficacy can be both categorical and quantitatively continuous. Thus the biomarkers can be either category classifier or quantitative indicator for the treatment status.

### Monitoring the drug toxicity

The first-line anti-TB drugs have been associated with toxicity, e.g., liver injury, for which the second-line drugs are presumably more severe. It is important to understand and monitor the drug toxicity, especially when long-term use is necessitated due to drug resistance of the pathogen. A biomarker for drug toxicity can be a quantitative indicator that measures the degree of damage to, say, the liver. This should be an important part of therapeutic monitoring. A limited few works are given in Table 5.

**TABLE 5:** Metabolomics biomarkers for drug toxicity.

Study	Sample/biofluids	Cohorts (size n)	Metabolome fraction	Analytical apparatus	Statistic methods	Metabolite biomarkers
Zhao et al. (2017)	Rat liver and serum	Rats (zero, low, high PZA dose) x (male, female), 10 each group	Total metobolome	^1^H NMR spectroscopy	PCA, OSC-PLS-DA, STT, non-parametric MWT, FDR	Increased LDL/VLDL, lactate, decreased BCAAs, glucose, taurine (high dose vs. zero dose)
Cao et al. (2018)	urine	TB pre-dose (25), TB-DILI (11, post-dose), non-DILI (49, post-dose)	total metobolome	UPLC-MS/MS	PCA, OPLS-DA, ANOVA test	pyroglutamate, isocitrate, citrate, xanthine; urate, cis-4-octenedioic acid, cis-aconitate, hypoxanthine
Deng et al. (2022)	Liver and serum bile acids, liver tissues	4 groups of mice (n = 8 each), treated with INH and/or RIF for 21 days	metabolome	LC-MS/MS	PLS-DA, one-way analysis of variance, Pearson’s test	CA, DCA, LCA, TDCA, TUDCA were potential biomarkers for early detection of RIF-induced liver injury

ANOVA = analysis of variance, BCAA = branched chains amino acids, CA = cholic acid, DCA = deoxycholic acid, FDR = hochberg and benjamini false discovery rate, INH = isoniazid, LCA = lithocholic acid, MWT = Mann–Whitney test, OPLS-DA = orthogonal PLS-DA, OSC-PLS-DA = orthogonal signal correction PLS-DA, PCA = principal component analysis, PLS-DA = partial least-squares discriminant analysis, PZA = pyrazinamide, (V) LDL = (very) low-density lipoprotein, STT = Student’s t-test, TDCA = taurodeoxycholic acid, TUDCA = tauroursodeoxycholic acid.

The first-line drugs usually induce hepatotoxicity. Indeed, PZA-induced hepatotoxicity was recently studied (Zhao et al., 2017) in rats *via* an NMR-based metabolomics approach complemented with histopathological analysis and clinical chemistry. PZA decreased the weights of dosed rats and induced dose-dependent liver injury, to which female rats were found more sensitive. It produced a status of oxidative stress and disturbances in purine, energy and nicotinamide adenine dinucleotide metabolisms in a gender-specific and dose-dependent manner. High dose of PZA caused increase of low-density lipoprotein/very low-density lipoprotein and lactate and decrease of glutamate, glycine, BCAAs, glucose, and taurine. Long-term or high-dose treatment with RIF can also induce severe liver injury. Using targeted bile acid (BA) metabolomics for four groups of mice, treated with different doses of INH and RIF, it was found (Deng et al., 2022) that RIF caused notable liver injury and increased serum cholic acid (CA) levels. Decline in the serum secondary BA levels led to liver injury in mice. CA, deoxycholic acid (DCA), lithocholic acid (LCA), taurodeoxycholic acid (TDCA), and tauroursodeoxycholic acid (TUDCA) were identified as potential biomarkers for early detection of RIF-induced liver injury. Furthermore, high dose RIF reduced hepatic BA levels and elevated serum BA levels.

Via urine metabolite profiling using UPLC-MS, the tricarboxylic acid circulation, arginine and proline metabolism and purine metabolic pathways were found to be affected by anti-TB drugs (Cao et al., 2018). The levels of pyroglutamate, isocitrate, citrate, and xanthine decreased significantly after drug treatment. In comparison between drug-induced liver injury (DILI) and non-DILI patients, urate and cis-4-octenedioic acid levels increased whereas the cis-aconitate and hypoxanthine levels decreased significantly, highlighting that superoxide generation can aggravate the hepatotoxic effects of the first drug regimen.

## Pathogen-based biomarkers

In this section the samples are mostly bacterial cultures and various Mtb strains as well as macrophages, rather than blood samples. The biomarkers in this section are associated with the pathogen, while in previous section, they are mainly associated with the host.

### Biomarkers for diagnosis and efficacy evaluation

The initial host-pathogen interaction, especially the capability of Mtb to utilize the carbon and nitrogen sources from the host to replicate, is crucial for the establishment of infection. Through the host-pathogen interaction, the metabolites of the pathogen enter the metabolic system of the hosts, which can become biomarkers for diagnosis and treatment monitoring as well as efficacy evaluation. Relevant works are listed in Table 6.

**TABLE 6:** Pathogen-based biomarkers for diagnosis and efficacy evaluation.

Study	Sample/biofluids	Cohorts (size n)	Metabolome fraction	Analytical apparatus	Statistic methods	Metabolite biomarkers
Ashokcoomar et al. (2020)	metabolites of bacterial strains	Wild type (10), ∆*mtp* mutant (10), *mtp*-complemented strains (10)	Metabolome, gene expression	GCxGC-MS	PCA, PLS-DA, *t*-test, Spearman’s test	28, 10, 16 significant differential metabolites were identified for each pair of strains.
Reedoy et al. (2020)	A549 epithelial cells	5 models comparing uninfected and infected WT, ∆*mtp, mtp*-complemented strains, and between strains.	Metabolome	GCxGC-MS	PCA, PLS-DA, *t*-test	Significant differential metabolomic profiles between WT- and ∆*mtp* infected A549 epithelial cell models of infection.
Ashokcoomar et al. (2021)	bacterial strains, THP-1 cells	WT- (9), Δ*mtp*- (8), complement-infected (8), Δ*mtp*- (9), complement-uninfected (9)	whole metabolome	GC×GC-MS	PCA, PLS-DA, FDR, *t-*test	MTP adhesin as a biomarker for diagnosis and therapeutic evaluation

FDR = hochberg and benjamini false discovery rate, MTP = mtb curli pili, PCA = principal component analysis, PLS-DA = partial least-squares discriminant analysis, WT = wild type.

Recently, Mtb curli pili (MTP) deficiency were found to be associated with alterations in cell wall biogenesis, fatty acid metabolism and amino acid synthesis (Ashokcoomar et al., 2020). Using untargeted GCxGC-MS, 28, 10, and 16 biologically significant differential metabolites were found between the *mtp* gene-knockout (∆*mtp*) mutants and the wild type (WT), between WT and *mtp-*complement, and between ∆*mtp* to *mtp-*complemented strains, respectively. This finding demonstrated that MTP can serve as a potential diagnostic biomarker. Another study (Reedoy et al., 2020) compared the metabolite profiles of A549 epithelial cells (with which MTP is associated with the host metabolism) between infected and uninfected, and between different strains of Mtb, and revealed significantly lower concentrations of 46 differential metabolites in the Δ*mtp*-infected cells, compared to WT-infected cells. In a THP-1 macrophage infection model (Ashokcoomar et al., 2021), MTP was found to be associated with alterations in carbon, fatty acid and amino acid metabolism. Metabolite profiling of THP-1 macrophages infected with the three types of strains revealed 9 and 10 significantly different metabolites in the Δmtp and complement strains, respectively, compared to the WT. The absence of the MTP adhesin resulted in reduced virulence of Mtb, suggesting the important role of MTP adhesin in modulating the host metabolic activity and as a promising biomarker for diagnosis and therapeutic evaluation.

Note that the numbers of differential metabolites could be reduced by other machine learning algorithms, e.g., RF and LASSO.

### Biomarkers for identifying drug resistance

Mtb displays a high degree of metabolic plasticity to adapt to the host environments. Genetic evidence suggests that Mtb relies mainly on fatty acid catabolism in the host. Thus, biomarkers could play an important role in discriminating drug resistant and drug sensitive Mtb (de Carvalho et al., 2010), as can be seen from the studies listed in Table 7.

**TABLE 7:** Pathogen-based biomarkers for drug resistance of Mtb.

Study	Sample/biofluids	Cohorts	Metabolome fraction	Analytical apparatus	Statistic methods	Metabolite biomarkers
Loots (2016)	Mtb strains, bacterial cultures	WT parent strain and mutants	MS from 50 to 800 m/z	GCxGC-MS	PCA, PLS-DA	22 biomarkers identified, including uridine, ribonic adic, aconitic acid, etc.
Lahiri et al. (2016)	Mtb strains, mutant cultures	Mtb mutants CDC1551and W-Beijing	lipidome	HPLC-MS	paired Student’s t test with multiple testing correction	mycobactin siderophores, carboxymycobactins, acylated sulfoglycolipids
Pal et al. (2017)	Mtb strains	Drug sensitive and resistant Mtb strains	lipidome	UPLC-MS	N/A	GPL, GMM (alpha-MA), GMM (methoxy-MA), DIM-B enhanced in DR, methoxy MA present only in DS Mtb
Nieto et al. (2018)	Mtb strains,	Clinical and laboratory clonal pair of Mtb strains, INHs and INHr	total metobolome, proteome	LC-MS/MS, TLC	Scaffold Local FDR algorithm, 2-tailed *t*-test	proteomic rearrangement and lipidomic changes after acquisition of INHr
Sun et al. (2019)	Mtb strains	MDR TB isolates before and 1 year after treatment, and H37Rv	total metobolome	LC-MS/MS	PCA, PLS-DA	175 differential metabolites, caused by 6 genetic mutations
Rêgo et al. (2021)	Mtb strains	53 Mtb strains (DS, MDR, XDR)	Total metabolome,	UPLC-MS, DI-MS	*t*-test, HCA	Isoleucine and proline levels higher in DS than DR strains

DI-MS = direct infusion mass spectrometry, DIM-B = dimycocerosate B, DR = drug resistant, DS = drug sensitive, FDR = hochberg and benjamini false discovery rate, GMM = glucose monomycolates, GPL = glycerophospholipids, HCA = hierarchical clustering analysis, INHr = isoniazid resistant, INHs = isoniazid susceptible, MA = mycolic acid, MDR = mulit-drug resistant, PCA = principal component analysis, PLS-DA = partial least-squares discriminant analysis, TLC = thin-layer chromatography, XDR = extensively drug resistant, WT = wild type.

Differential fatty acyls and glycerophospholipids (GPLs) were observed in the lipidome profiles in a comparative lipidomics study (Pal et al., 2017) between drug sensitive (DS) and DR strains of Mtb. GPLs, glucose monomycolates (alpha mycolic acid, methoxy mycolic acid) and dimycocerosate B were found enhanced in DR Mtb while methoxy mycolic acid was present only in DS Mtb. These different lipids may serve as a resource for identifying biomarkers aimed at disrupting the functions of Mtb lipids associated with drug resistance. Using UPLC-MS-based metabolomics, Rêgo et al. (2021) reported that DS, MDR and XDR Mtb strains had distinct metabolic profiles, which could be used to predict drug susceptibility and resistance. It was found that levels of isoleucine and proline, as well as ions presumptively identified as hercynine, betaine, and pantothenic acid, varied significantly between these strains. In particular, the levels of isoleucine and proline were significantly higher in DS strains compared to MDR and XDR strains, and thus may serve as distinguishing biomarkers.

A few studies focused on the resistance to specific drugs. *via* a 2D GC-MS metabolomics approach, Loots (2016) reported that 22 biomarkers could be used to characterize RR Mtb. These biomarkers indicated an instability in mRNA of RR Mtb and a total depletion of aconitic acid and a subsequently increased dependency on alternative energy sources. Other metabolic changes were associated with a survival response for maintaining/remodeling the cell wall. An unbiased MS-based organism-wide lipidomic profiling (Lahiri et al., 2016) revealed that RR mutations led to altered concentrations of mycobactin siderophores and acylated sulfoglycolipids, providing direct evidence for characteristic remodeling of cell wall lipids in RR strains of Mtb, as well as evidence that the RR RpoB mutations were associated with a reduction in sulfoglycolipids. A recent proteomics and lipidomics-based study (Nieto et al., 2018) focused on biochemical characterization of INH resistant Mtb using clonal pairs of clinical and laboratory-generated strains, and found 26 Mtb proteins with altered abundances and lipidome changes after acquisition of INH resistance across both Mtb genetic lineages studied. It was recently shown that genetic mutations of Mtb induced by anti-TB treatment led to metabolism changes and elevation of EMB resistance (Sun et al., 2019). A total of 175 differential metabolites were identified, caused by six genetic mutations after anti-TB treatment. They were mainly involved in amino sugar and nucleotide sugar metabolism, β-alanine metabolism, sulfur metabolism, and galactose metabolism. It would be highly desirable to dramatically reduce the number of key differential metabolites.

## Discussion

Owing to its huge negative impact, TB has been under intensive and extensive studies, to which the modern omics technologies have been widely applied. Over the past few years, significant progress has been made in terms of biomarker discovery using metabolomics and lipidomics. Various potential biomarkers have been identified, especially diagnostic biomarkers. Many issues and limitations remain, however. In particular, the clinical potentiality of these biomarkers requires future validation.

**FIGURE 2:**
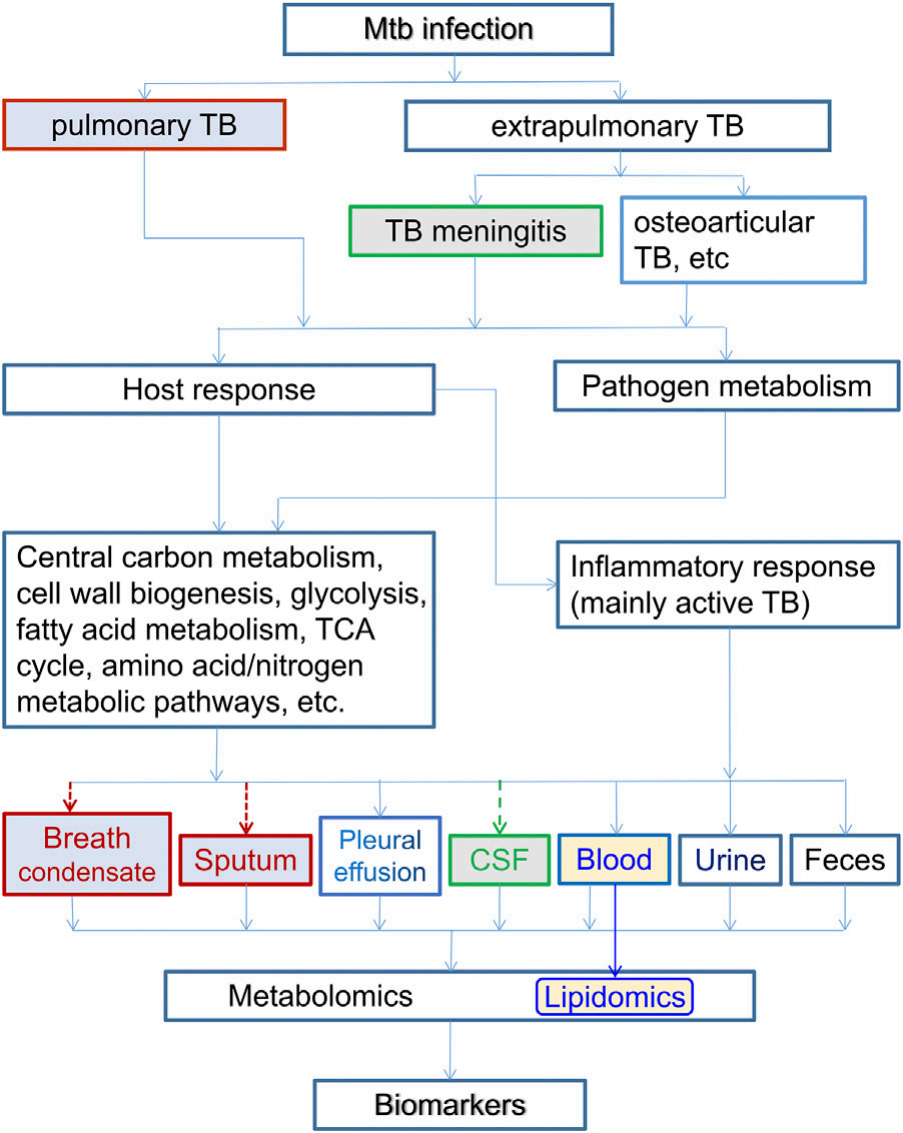
The relation of host-based metabolomics and lipidomics branch with various types of TB and associated samples. Here the inflammatory response are associated with host immune system of mostly active TB patients. Both the host and pathogen metabolisms cause dysregulation and metabolomic changes, which are reflected in various body fluids. Breath condensate and sputum are for pulmonary TB only, and CSF is for TBM only. Lipidomics usually uses blood samples. All types of body fluids can be samples for metabolomics in general.

We have discussed recent studies in pulmonary and extrapulmonary TB, active TB and LTBI, using different types of samples for metabolomics, including breath condensate, sputum, pleural effusion, blood, CSF, urine, and fecal samples (Figure 2). In particular, CSF is used for TBM only, and breath condensate and sputum are relevant mostly for pulmonary TB, even though they may carry metabolites of extrapulmonary TB as well. Although both host response to Mtb infection and pathogen metabolism cause changes in the metabolome, pro- and anti-inflammatory responses are associated with the immune system of mainly active TB patients, leading to dysregulation of cytokines and associate metabolites involved in relevant metabolic pathways. Despite of being asymptomatic, LTBI may also to certain degrees lead to immunological response and hence associated metabolic dysregulation, even though the symptom is not apparent. Apart from the inflammatory response, the main host metabolic changes are associated with glycolysis and glyconeogenesis, TCA cycle, urea cycle, pentose phosphate pathways, lipids and amino acids metabolism, iron metabolism, and cerebral tryptophan metabolism (for TBM), tryptophan/kynurenine pathway, etc. The central carbon metabolism of Mtb involves glycolysis, TCA cycle, glyoxylate shunt, methylcitrate cycle, pentose phosphate pathway, bile acid biosynthesis, etc. The identified biomarker metabolites participate in various biological processes. For example, pyruvate and lactate are involved in glycolysis, itaconate, aconitic acid and succinate are involved in the TCA cycle, arginine, citruline, tryptophan, kynurenine, glutamine and various PCs, PEs, PIs, SMs, etc., are involved in lipid and amino acid metabolism. Some metabolites, e.g., amino acids, are involved in multiple pathways, resulting in a complex metabolic pathway network. Also some metabolites, e.g., betaine, are involved in the pathogens−host interaction during Mtb infection.

A great number of potential biomarker metabolites have been discovered either for diagnosis of TB, or for distinguishing TB patients from non-TB controls, including HCs, LTBI/HHCs and other diseases that may have similar symptoms. Most of these studies focused on pulmonary TB, which is the prevalent form of tuberculosis, while a few others studied TBM, mostly based on CSF or urine samples. In the meantime, limited studies focused on biomarkers for identifying LTBI, since most studies would naturally include TB patients in the cohorts.

Monitoring the efficacy of medical therapy and the progress of treatment is an important aspect of the entire treatment process, especially when therapy adjustment is needed for long-term treatments. It is also important to avoid premature release of TB patients. To avoid the month-long waiting time for sputum culturing, blood, urine and/or fecal samples are usually used for all TB types, pleural effusions and exhaled breath condensate for pulmonary TB, and CSF samples for TBM. These studies usually involve humans as the subjects, with occasional use of non-human animal models. A large number of differential metabolites are associated with altered energy metabolism, immunophysiology, pathogen cell wall disruption and repair.

A few works identified biomarkers for drug toxicity, which is in fact a very important issue, as it can prevent long-term or high-dose administration of a drug that causes severe side effect damages, and thus should be part of therapeutic monitoring.

The experimental subjects were switched mostly from human to pathogen strains and host macrophages in the metabolomic studies on drug resistance. The laboratory strain, Mtb H37Rv and its mutant variations as well as clinical isolates are the most commonly used samples. However, supervised machine learning was rarely used in these studies to further select biomarkers from a large number of differential metabolites.

There have been many studies on the drug resistance of the pathogen. Anti-TB drugs normally act by inhibiting various enzymes associated with central carbon and nitrogen metabolism and biosynthesis for cell wall growth. In reaction, the pathogen can adapt to the micro environment inside the macrophage to shunt antibiotics. It can adaptively remodel its metabolic network for repair of the cell wall and DNA damages. Furthermore, it may develop drug resistance in response to various types of stress mostly by entering a dormant state with very low level of metabolic activity and/or by cell wall lipid reorganization. As a result, there are distinct differences in metabolic profile between DS and DR, especially MDR and XDR, strains of Mtb.

Despite the great progress that has been made, there are apparent limitations in these studies. 1) Most studies were based on small cohorts, which impaired the statistical reliability of the findings; some typical bioinformatics or machine learning algorithms require a minimum of 30 samples to yield a meaningful result. 2) Lacking a uniform standard, the experimental conditions and analytical apparatuses differ from one study to another. (This is a roadblock that can be removed *via* technological upgrade.) 3) Most biomarkers were not independently validated. 4) Many studies found a large number of biomarkers, only based on PCA or PLS-DA, or simply FC and *p*-value screening. More elaborated selections based on better supervised machine learning algorithms, such as LASSO and RF, are needed, in order to identify key differential metabolites with the best performance and clinical applicability. 5) The statistical differentiation in the metabolome between different cohorts was only marginal in some of the studies, as revealed by the overlap in PCA and/or PLS-DA plots. 6) The reported potential biomarkers differ greatly from one study to another. *Only a very limited number of potential diagnostic biomarkers appeared in more than one studies,* including glutamate, glutamine, methionine, creatine, cysteine, tryptophan, threonine, citrate, citrulline, and citric acid. 7) Due to the large disparity in experimental design (and experimental conditions), it is, at present, hard to compare (and select) the biomarkers between different studies. Therefore, there is still a long way to go before these biomarkers are fully validated for clinical application.

**FIGURE 3:**
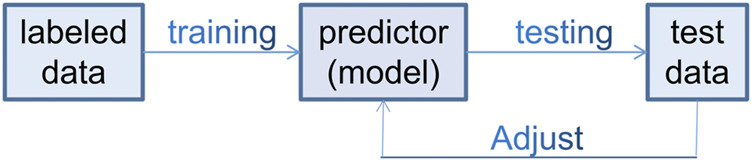
Schematic flow diagram of typical supervised machine learning in biomarker discovery.

We emphasize that machine learning has become increasingly more important in biomarker discovery. It has been widely used across different disciplines and fields, from fundamental basic science (Chmiela et al., 2020) to industrial applications (Datta and Davim, 2022). While the usual statistical methods of PCA, HCA and K mean clustering have been used in unsupervised machine learning, what is particularly useful for biomarker discovery is supervised machine learning. The widely used (O)PLS-DA is a supervised dimension-reduction procedure that finds new features that not only captures most of the information in the original variables, but also are related to the response (Boehmke and Greenwell, 2020). However, a study (Westerhuis et al., 2008) showed that PLS-DA score plots often present an *overoptimistic* view of the class separation. In general, supervised machine learning takes labeled data as input to train the model, using certain algorithms, such as LASSO, RF, SVM, and artificial neural network, and then test the model with new data and adjust the model as needed (Jung, 2022) (Figure 3). It is particularly useful in feature selection and classification, and thus suitable for biomarker discovery as the typical data labels are categorical. In comparison with (O)PLS-DA, LASSO and RF, for example, are capable of avoiding the over-fitting and multi-collinearity issues that are often present in the omics data, thus can select among a large number of differential metabolites (features) the most important ones as the biomarkers. However, machine learning often takes a large amount of data to train the model (Beleites et al., 2013). For example, it was reported that modern modeling techniques such as SVM, neural network and RF may need over 200 events (TB patients) per variable (biomarker) (van der Ploeg et al., 2014). A manifestation of small sample size effect is the presence of step jumps in the ROC curves. As the threshold varies, the false positive changes one by one, leading to such jumps when the sample size is small.

A few studies discussed above involved a fairly large number of samples, especially those for the identification of LTBI. Possibly because the metabolic dysregulations in HHCs were intrinsically not strong enough, these studies did not find very strong metabolomic biomarkers. A couple of studies involved over 100 samples in each cohorts. Isa et al. (2018) used 107 TB patients with 102 asymptomatic controls, and identified four potential biomarkers using RF. Luo Y. et al (2020) had 125 TB patients and 101 LTBI, however, the differences were investigated only with the Mann–Whitney *U* test or Chi-square test. Among all studies discussed above, a small percentage used both discovery and validation cohorts, but most of them used only PCA, HCA, and PLS-DA analysis. Non-etheless, a small fraction did use more sophisticated machine learning algorithms, such as LASSO, RF, SVM, and elastic net model. It will be highly desirable to combine large sample size with advanced machine learning algorithms.

One important issue is why the biomarkers from different studies seldom overlap, even when the groups to be compared are the same, say, TB versus HCs? This happens between different research teams and also between different studies of the same teams. One can in part blame the small sample size and statistical fluctuations in individual systemic biological state. However, a fundamental question is, are different untargeted metabolomic studies supposed to yield the same set of biomarkers in ideal conditions? Alternatively, if one manages to have an infinitely large data set, say, of TB vs. HCs, spanning all other possible experimental parameters, should there be at least one metabolite that is consistently upregulated (or downregulated) in all TB samples as compared to HCs? An implicit guiding principle behind all the biomarker research is that the answer is Yes. However, one must consider the possibility that there are multiple possible independent systemic metabolic states that manifest the same typical TB symptoms, yet none of the metabolites is consistently up- or down-regulated across different states, even though there are consistent biomarkers for each of these pathological states as compared with HCs. Just as for a non-linear complex equation, there often exist multiple solutions. This is certainly possible given the potentially huge individual disparity, and should be kept in mind in future researches.

Looking forward, there are two obvious research directions. One is to design new studies to find more biomarkers. The other is to re-examine the already discovered potential biomarkers with new experiments. This makes sense in that these biomarkers already cover basically all major metabolic pathways. Only when a biomarker (or a panel of biomarkers) is validated by multi-studies can it be applied for clinical application. There is also a third direction. Given the data sharing service in open scientific data repositories, it is possible to (re) do data analysis with possibly combined data using more advanced machine learning algorithms. In fact, some biomarkers from different studies are associated with same metabolic pathways. These studies have a high potential to find common biomarkers once the sample sets are enlarged. With machine learning plus big data, it is hopeful that commonly recognized biomarkers will be found.

Finally, we mention that it helps to integrate metabolomics with other omics approaches, including proteomics, transcriptomics and genomics, as already done in some of the studies discussed above, in order to find better biomarkers for TB.

## Conclusion

In summary, great progress has been made in biomarker discovery for diagnosis and treatment monitoring of TB based on metabolomics over the past several years. Further validation are of utmost importance prior to application of these metabolome-based biomarkers in the clinical setting. Overall, a large number of differential metabolites or biomarker candidates have been found. However, improved bioinformatics data analyses are needed, to pin down key biomarkers from a large number of differential metabolites. This warrants more intensive and extensive future research effort in this area, with the help of machine learning, in the hope that clinically applicable biomarkers will be made available in the near future.

## References

[B1] Albors-Vaquer A. Rizvi A. Matzapetakis M. Lamosa P. Coelho A. V. Patel A. B. (2020). Active and prospective latent tuberculosis are associated with different metabolomic profiles: Clinical potential for the identification of rapid and non-invasive biomarkers. Emerg. Microbes Infect. 9, 1131–1139. 10.1080/22221751.2020.1760734 32486916 PMC7448900

[B3] Ashokcoomar S. Loots D. T. Beukes D. van Reenen M. Pillay B. Pillay M. (2021). *M. tuberculosis* curli pili (MTP) is associated with alterations in carbon, fatty acid and amino acid metabolism in a THP-1 macrophage infection model. Microb. Pathog. 154, 104806. 10.1016/j.micpath.2021.104806 33610716

[B4] Ashokcoomar S. Reedoy K. S. Senzani S. Loots D. T. Beukes D. van Reenen M. (2020). *Mycobacterium tuberculosis* curli pili (MTP) deficiency is associated with alterations in cell wall biogenesis, fatty acid metabolism and amino acid synthesis. Metabolomics 16, 97. 10.1007/s11306-020-01720-z 32914199

[B5] Awasthi D. Freundlich J. S. (2017). Antimycobacterial metabolism: Illuminating *Mycobacterium tuberculosis* biology and drug discovery. Trends Microbiol. 25, 756–767. 10.1016/j.tim.2017.05.007 28622844 PMC5564221

[B6] Bacakoğlu F. Başoğlu Ö. K. Çok G. Sayıner A. Ateş M. (2001). Pulmonary tuberculosis in patients with diabetes mellitus. Respiration 68, 595–600. 10.1159/000050578 11786714

[B7] Beccaria M. Bobak C. Maitshotlo B. Mellors T. R. Purcaro G. Franchina F. A. (2018). Exhaled human breath analysis in active pulmonary tuberculosis diagnostics by comprehensive gas chromatography-mass spectrometry and chemometric techniques. J. Breath. Res. 13, 016005. 10.1088/1752-7163/AAE80E 30394364 PMC6537098

[B8] Beleites C. Neugebauer U. Bocklitz T. Krafft C. Popp J. (2013). Sample size planning for classification models. Anal. Chim. Acta 760, 25–33. 10.1016/j.aca.2012.11.007 23265730

[B9] Biadglegne F. Schmidt J. R. Engel K. M. Lehmann J. Lehmann R. T. Reinert A. (2022). *Mycobacterium tuberculosis* affects protein and lipid content of circulating exosomes in infected patients depending on tuberculosis disease state. Biomedicines 10, 783. 10.3390/biomedicines10040783 35453532 PMC9025801

[B10] Boehme C. C. Nabeta P. Hillemann D. Nicol M. P. Shenai S. Krapp F. (2010). Rapid molecular detection of tuberculosis and rifampin resistance. N. Engl. J. Med. 363, 1005–1015. 10.1056/NEJMoa0907847 20825313 PMC2947799

[B11] Boehmke B. Greenwell B. (2020). Hands-on machine learning with R. Boca Raton: CRC Press.

[B12] Brandenburg J. Heyckendorf J. Marwitz F. Zehethofer N. Linnemann L. Gisch N. (2022). Tuberculostearic acid-containing phosphatidylinositols as markers of bacterial burden in tuberculosis. ACS Infect. Dis. 8, 1303–1315. 10.1021/ACSINFECDIS.2C00075 35763439 PMC9274766

[B13] Cao J. Mi Y. Shi C. Bian Y. Huang C. Ye Z. (2018). First-line anti-tuberculosis drugs induce hepatotoxicity: A novel mechanism based on a urinary metabolomics platform. Biochem. Biophys. Res. Commun. 497, 485–491. 10.1016/j.bbrc.2018.02.030 29454961

[B14] Chandra P. Coullon H. Agarwal M. Goss C. W. Philips J. A. (2022). Macrophage global metabolomics identifies cholestenone as host/pathogen cometabolite present in human *Mycobacterium tuberculosis* infection. J. Clin. Invest. 132, e152509. 10.1172/JCI152509 35104812 PMC8803325

[B15] Che N. Cheng J. Li H. Zhang Z. Zhang X. Ding Z. (2013). Decreased serum 5-oxoproline in TB patients is associated with pathological damage of the lung. Clin. Chim. Acta 423, 5–9. 10.1016/J.CCA.2013.04.010 23608301

[B16] Che N. Ma Y. Ruan H. Xu L. Wang X. Yang X. (2018). Integrated semi-targeted metabolomics analysis reveals distinct metabolic dysregulation in pleural effusion caused by tuberculosis and malignancy. Clin. Chim. Acta 477, 81–88. 10.1016/j.cca.2017.12.003 29208371

[B17] Chen D. Bryden W. A. Wood R. (2020). Detection of tuberculosis by the analysis of exhaled breath particles with high-resolution mass spectrometry. Sci. Rep. 10, 7647. 10.1038/s41598-020-64637-6 32376992 PMC7203136

[B18] Chen J.-X. Han Y.-S. Zhang S.-Q. Li Z.-B. Chen J. Yi W.-J. (2021). Novel therapeutic evaluation biomarkers of lipid metabolism targets in uncomplicated pulmonary tuberculosis patients. Signal Transduct. Target. Ther. 6, 22. 10.1038/s41392-020-00427-w 33462176 PMC7814055

[B19] Chen X. Ye J. Lei H. Wang C. (2022). Novel potential diagnostic serum biomarkers of metabolomics in osteoarticular tuberculosis patients: A preliminary study. Front. Cell. Infect. Microbiol. 12, 827528. 10.3389/fcimb.2022.827528 35402287 PMC8992656

[B20] Chmiela S. Sauceda H. E. Tkatchenko A. Müller K.-R. (2020). in Machine learning meets quantum physics. Editors Schütt K. T. Chmiela S. von Lilienfeld O. A. Tkatchenko A. Tsuda K. Müller K.-R. (Cham: Springer International Publishing). 10.1007/978-3-030-40245-7

[B21] Cho Y. Park Y. Sim B. Kim J. Lee H. Cho S.-N. (2020). Identification of serum biomarkers for active pulmonary tuberculosis using a targeted metabolomics approach. Sci. Rep. 10, 3825. 10.1038/s41598-020-60669-0 32123207 PMC7052258

[B22] Collins J. M. Jones D. P. Sharma A. Khadka M. Liu K. H. Kempker R. R. (2021). TCA cycle remodeling drives proinflammatory signaling in humans with pulmonary tuberculosis. PLOS Pathog. 17, e1009941. 10.1371/JOURNAL.PPAT.1009941 34559866 PMC8494353

[B23] Collins J. M. Siddiqa A. Jones D. P. Liu K. Kempker R. R. Nizam A. (2020). Tryptophan catabolism reflects disease activity in human tuberculosis. JCI Insight 5, e137131. 10.1172/jci.insight.137131 32369456 PMC7259525

[B24] Collins J. M. Walker D. I. Jones D. P. Tukvadze N. Liu K. H. Tran V. T. (2018). High-resolution plasma metabolomics analysis to detect Mycobacterium tuberculosis-associated metabolites that distinguish active pulmonary tuberculosis in humans. PLoS One 13, e0205398. 10.1371/JOURNAL.PONE.0205398 30308073 PMC6181350

[B25] Combrink M. Du Preez I. Ronacher K. Walzl G. Loots D. T. (2019). Time-dependent changes in urinary metabolome before and after intensive phase tuberculosis therapy: A pharmacometabolomics study. Omi. A J. Integr. Biol. 23, 560–572. 10.1089/OMI.2019.0140 31613685

[B26] Combrink M. Loots D. T. du Preez I. (2020). Metabolomics describes previously unknown toxicity mechanisms of isoniazid and rifampicin. Toxicol. Lett. 322, 104–110. 10.1016/J.TOXLET.2020.01.018 31981687

[B27] Comella-del-Barrio P. Izquierdo-Garcia J. L. Gautier J. Doresca M. J. C. Campos-Olivas R. Santiveri C. M. (2021). Urine NMR-based TB metabolic fingerprinting for the diagnosis of TB in children. Sci. Rep. 11, 12006. 10.1038/s41598-021-91545-0 34099838 PMC8184981

[B28] Dai Y.-N. Huang H.-J. Song W.-Y. Tong Y.-X. Yang D.-H. Wang M.-S. (2017). Identification of potential metabolic biomarkers of cerebrospinal fluids that differentiate tuberculous meningitis from other types of meningitis by a metabolomics study. Oncotarget 8, 100095–100112. 10.18632/ONCOTARGET.21942 29245963 PMC5725005

[B29] Das M. K. Arya R. Debnath S. Debnath R. Lodh A. Bishwal S. C. (2016). Global urine metabolomics in patients treated with first-line tuberculosis drugs and identification of a novel metabolite of ethambutol. Antimicrob. Agents Chemother. 60, 2257–2264. 10.1128/AAC.02586-15 26833163 PMC4808164

[B31] Datta S. Davim J. P. (2022). in Machine learning in industry. Editors Datta S. Davim J. P. (Cham: Springer International Publishing). 10.1007/978-3-030-75847-9

[B32] de Carvalho L. P. S. Fischer S. M. Marrero J. Nathan C. Ehrt S. Rhee K. Y. (2010). Metabolomics of *Mycobacterium tuberculosis* reveals compartmentalized Co-catabolism of carbon substrates. Chem. Biol. 17, 1122–1131. 10.1016/j.chembiol.2010.08.009 21035735

[B33] Deng J. Liu L. Yang Q. Wei C. Zhang H. Xin H. (2021). Urinary metabolomic analysis to identify potential markers for the diagnosis of tuberculosis and latent tuberculosis. Arch. Biochem. Biophys. 704, 108876. 10.1016/j.abb.2021.108876 33864753

[B34] Deng Y. Luo X. Li X. Xiao Y. Xu B. Tong H. (2022). Screening of biomarkers and toxicity mechanisms of rifampicin-induced liver injury based on targeted bile acid metabolomics. Front. Pharmacol. 13, 925509. 10.3389/fphar.2022.925509 35754491 PMC9226894

[B35] Diboun I. Cyprian F. S. Anwardeen N. R. Yassine H. M. Elrayess M. A. Rahmoon S. M. (2022). Identification of prognostic metabolomic biomarkers at the interface of mortality and morbidity in pre-existing TB cases infected with SARS-CoV-2. Front. Cell. Infect. Microbiol. 12, 929689. 10.3389/fcimb.2022.929689 35937683 PMC9354137

[B36] Ding Y. Raterink R.-J. Marín-Juez R. Veneman W. J. Egbers K. van den Eeden S. (2020). Tuberculosis causes highly conserved metabolic changes in human patients, mycobacteria-infected mice and zebrafish larvae. Sci. Rep. 10, 11635. 10.1038/s41598-020-68443-y 32669636 PMC7363909

[B37] du Preez I. Luies L. Loots D. T. (2017). Metabolomics biomarkers for tuberculosis diagnostics: Current status and future objectives. Biomark. Med. 11, 179–194. 10.2217/bmm-2016-0287 28097879

[B38] du Preez I. Luies L. Loots D. T. (2019). The application of metabolomics toward pulmonary tuberculosis research. Tuberculosis 115, 126–139. 10.1016/J.TUBE.2019.03.003 30948167

[B39] Duffy F. J. Weiner J. Hansen S. Tabb D. L. Suliman S. Thompson E. (2019). Immunometabolic signatures predict risk of progression to active tuberculosis and disease outcome. Front. Immunol. 10, 527. 10.3389/fimmu.2019.00527 30967866 PMC6440524

[B40] Dutta N. K. Tornheim J. A. Fukutani K. F. Paradkar M. Tiburcio R. T. Kinikar A. (2020). Integration of metabolomics and transcriptomics reveals novel biomarkers in the blood for tuberculosis diagnosis in children. Sci. Rep. 10, 19527. 10.1038/s41598-020-75513-8 33177551 PMC7658223

[B41] Fitzgerald B. L. Islam M. N. Graham B. Mahapatra S. Webb K. Boom W. H. (2019). Elucidation of a human urine metabolite as a seryl-leucine glycopeptide and as a biomarker of effective anti-tuberculosis therapy. ACS Infect. Dis. 5, 353–364. 10.1021/acsinfecdis.8b00241 30585483 PMC6412501

[B42] Frediani J. K. Jones D. P. Tukvadze N. Uppal K. Sanikidze E. Kipiani M. (2014). Plasma metabolomics in human pulmonary tuberculosis disease: A pilot study. PLoS One 9, e108854. 10.1371/journal.pone.0108854 25329995 PMC4198093

[B43] Goff A. Cantillon D. Muraro Wildner L. Waddell S. J. (2020). Multi-omics technologies applied to tuberculosis drug discovery. Appl. Sci. 10, 4629. 10.3390/app10134629

[B44] Goletti D. Lee M. R. Wang J. Y. Walter N. Ottenhoff T. H. M. (2018). Update on tuberculosis biomarkers: From correlates of risk, to correlates of active disease and of cure from disease. Respirology 23, 455–466. 10.1111/RESP.13272 29457312

[B45] Goletti D. Petruccioli E. Joosten S. A. Ottenhoff T. H. M. (2016). Tuberculosis biomarkers: From diagnosis to protection. Infect. Dis. Rep. 8, 6568. 10.4081/idr.2016.6568 27403267 PMC4927936

[B47] Haas C. T. Roe J. K. Pollara G. Mehta M. Noursadeghi M. (2016). Diagnostic ‘omics’ for active tuberculosis. BMC Med. 14, 37. 10.1186/s12916-016-0583-9 27005907 PMC4804573

[B48] Han Y. S. Chen J. X. Li Z. B. Chen J. Yi W. J. Huang H. (2021). Identification of potential lipid biomarkers for active pulmonary tuberculosis using ultra-high-performance liquid chromatography-tandem mass spectrometry. Exp. Biol. Med. 246, 387–399. 10.1177/1535370220968058 PMC7885049 33175608

[B49] Hu X. Wang J. Ju Y. Zhang X. Qimanguli W. Li C. (2022). Combining metabolome and clinical indicators with machine learning provides some promising diagnostic markers to precisely detect smear-positive/negative pulmonary tuberculosis. BMC Infect. Dis. 22, 707. 10.1186/s12879-022-07694-8 36008772 PMC9403968

[B50] Huang H. Shi L. Y. Wei L. L. Han Y. S. Yi W. J. Pan Z. W. (2019). Plasma metabolites Xanthine, 4-Pyridoxate, and d-glutamic acid as novel potential biomarkers for pulmonary tuberculosis. Clin. Chim. Acta 498, 135–142. 10.1016/J.CCA.2019.08.017 31442449

[B51] Huynh J. Donovan J. Phu N. H. Nghia H. D. T. Thuong N. T. T. Thwaites G. E. (2022). Tuberculous meningitis: Progress and remaining questions. Lancet Neurol. 21, 450–464. 10.1016/S1474-4422(21)00435-X 35429482

[B52] Isa F. Collins S. Lee M. H. Decome D. Dorvil N. Joseph P. (2018). Mass spectrometric identification of urinary biomarkers of pulmonary tuberculosis. EBioMedicine 31, 157–165. 10.1016/J.EBIOM.2018.04.014 29752217 PMC6013777

[B53] Isaiah S. Loots D. T. Solomons R. van der Kuip M. Tutu Van Furth A. M. Mason S. (2020). Overview of brain-to-gut Axis exposed to chronic CNS bacterial infection(s) and a predictive urinary metabolic profile of a brain infected by *Mycobacterium tuberculosis* . Front. Neurosci. 14, 296. 10.3389/FNINS.2020.00296 32372900 PMC7186443

[B54] Izquierdo-Garcia J. L. Comella-del-Barrio P. Campos-Olivas R. Villar-Hernández R. Prat-Aymerich C. De Souza-Galvão M. L. (2020). Discovery and validation of an NMR-based metabolomic profile in urine as TB biomarker. Sci. Rep. 10, 22317. 10.1038/s41598-020-78999-4 33339845 PMC7749110

[B55] Jansen R. S. Rhee K. Y. (2017). Emerging approaches to tuberculosis drug development: At home in the metabolome. Trends Pharmacol. Sci. 38, 393–405. 10.1016/j.tips.2017.01.005 28169001 PMC5367985

[B56] Jiang J. Li Z. Chen C. Jiang W. Xu B. Zhao Q. (2021). Metabolomics strategy assisted by transcriptomics analysis to identify potential biomarkers associated with tuberculosis. Infect. Drug Resist. 14, 4795–4807. 10.2147/IDR.S330493 34815677 PMC8604652

[B57] Jiang L. Wang J. Xu L. Cai J. Zhao S. Ma A. (2022). Lactobacillus casei modulates inflammatory cytokines and metabolites during tuberculosis treatment: A post hoc randomized controlled trial. Asia Pac. J. Clin. Nutr. 31, 66–77. 10.6133/APJCN.202203_31(1).0008 35357105

[B59] Jung A. (2022). Machine learning. Singapore: Springer Nature Singapore. 10.1007/978-981-16-8193-6

[B60] Kontsevaya I. Lange C. Comella-del-Barrio P. Coarfa C. DiNardo A. R. Gillespie S. H. (2021). Perspectives for systems biology in the management of tuberculosis. Eur. Respir. Rev. 30, 200377. 10.1183/16000617.0377-2020 34039674 PMC9488731

[B61] Krishnan S. Queiroz A. T. L. Gupta A. Gupte N. Bisson G. P. Kumwenda J. (2021). Integrative multi-omics reveals serum markers of tuberculosis in advanced HIV. Front. Immunol. 12, 676980. 10.3389/fimmu.2021.676980 34168648 PMC8217878

[B62] Kumar N. Shreshtha A. K. Patra S. (2017). The metabolomic strategy in tuberculosis therapy. Comb. Chem. High. Throughput Screen 20, 235–246. 10.2174/1386207320666170309111135 28290241

[B63] Lahiri N. Shah R. R. Layre E. Young D. Ford C. Murray M. B. (2016). Rifampin resistance mutations are associated with broad chemical remodeling of *Mycobacterium tuberculosis* . J. Biol. Chem. 291, 14248–14256. 10.1074/JBC.M116.716704 27226566 PMC4933180

[B64] Li Z. Du B. Zheng X. Jia H. Xing A. Sun Q. (2017). Cerebrospinal fluid metabolomic profiling in tuberculous and viral meningitis: Screening potential markers for differential diagnosis. Clin. Chim. Acta 466, 38–45. 10.1016/J.CCA.2017.01.002 28063937

[B65] Liebenberg C. Luies L. Williams A. A. (2021). Metabolomics as a tool to investigate HIV/TB Co-infection. Front. Mol. Biosci. 8, 692823. 10.3389/fmolb.2021.692823 34746228 PMC8565463

[B66] Liu J. Tang L. Lu Q. Yu Y. Xu Q.-G. Zhang S. (2022). Plasma quantitative lipid profiles: Identification of CarnitineC18:1-OH, CarnitineC18:2-OH and FFA (20:1) as novel biomarkers for pre-warning and prognosis in acute myocardial infarction. Front. Cardiovasc. Med. 9, 848840. 10.3389/fcvm.2022.848840 35479277 PMC9037999

[B67] Liu Y. Mei B. Chen D. Cai L. (2021). GC‐MS metabolomics identifies novel biomarkers to distinguish tuberculosis pleural effusion from malignant pleural effusion. J. Clin. Lab. Anal. 35, e23706. 10.1002/jcla.23706 33528039 PMC8059743

[B68] Loots D. T. (2016). New insights into the survival mechanisms of rifampicin-resistant *Mycobacterium tuberculosis* . J. Antimicrob. Chemother. 71, 655–660. 10.1093/JAC/DKV406 26679254

[B69] López-Hernández Y. Lara-Ramírez E. E. Salgado-Bustamante M. López J. A. Oropeza-Valdez J. J. Jaime-Sánchez E. (2019). Glycerophospholipid metabolism alterations in patients with type 2 diabetes mellitus and tuberculosis comorbidity. Arch. Med. Res. 50, 71–78. 10.1016/J.ARCMED.2019.05.006 31349956

[B70] Luier L. Loots D. T. (2016). Tuberculosis metabolomics reveals adaptations of man and microbe in order to outcompete and survive. Metabolomics 12, 40. 10.1007/s11306-016-0969-x

[B71] Luies L. du Preez I. Loots D. T. (2017a). The role of metabolomics in tuberculosis treatment research. Biomark. Med. 11, 1017–1029. 10.2217/bmm-2017-0141 29039217

[B72] Luies L. Mienie J. Motshwane C. Ronacher K. Walzl G. Loots D. T. (2017b). Urinary metabolite markers characterizing tuberculosis treatment failure. Metabolomics 13, 124. 10.1007/s11306-017-1261-4

[B73] Luies L. Reenen M. V. Ronacher K. Walzl G. Loots D. T. (2017c). Predicting tuberculosis treatment outcome using metabolomics. Biomark. Med. 11, 1057–1067. 10.2217/bmm-2017-0133 29172670

[B74] Luo P. Mao K. Xu J. Wu F. Wang X. Wang S. (2020a). Metabolic characteristics of large and small extracellular vesicles from pleural effusion reveal biomarker candidates for the diagnosis of tuberculosis and malignancy. J. Extracell. Vesicles 9, 1790158. 10.1080/20013078.2020.1790158 32944177 PMC7480510

[B75] Luo Y. Xue Y. Lin Q. Tang G. Yuan X. Mao L. (2020b). A combination of iron metabolism indexes and tuberculosis-specific antigen/phytohemagglutinin ratio for distinguishing active tuberculosis from latent tuberculosis infection. Int. J. Infect. Dis. 97, 190–196. 10.1016/j.ijid.2020.05.109 32497795

[B76] Magdalena D. Michal S. Marta S. Magdalena K.-P. Anna P. Magdalena G. (2022). Targeted metabolomics analysis of serum and *Mycobacterium tuberculosis* antigen-stimulated blood cultures of pediatric patients with active and latent tuberculosis. Sci. Rep. 12, 4131. 10.1038/s41598-022-08201-4 35260782 PMC8904507

[B77] Mason S. Reinecke C. J. Solomons R. (2017). Cerebrospinal fluid amino acid profiling of pediatric cases with tuberculous meningitis. Front. Neurosci. 11, 534. 10.3389/fnins.2017.00534 29018323 PMC5623012

[B78] Mason S. van Furth A. M. T. Solomons R. Wevers R. A. van Reenen M. Reinecke C. J. (2016). A putative urinary biosignature for diagnosis and follow-up of tuberculous meningitis in children: Outcome of a metabolomics study disclosing host–pathogen responses. Metabolomics 12, 110. 10.1007/s11306-016-1053-2

[B79] Meng R. Dong W. Gao J. Lu C. Zhang C. Liao Q. (2022). *Clostridium*, *Bacteroides* and *Prevotella* associates with increased fecal metabolites *Trans*-4-Hydroxy-L-proline and Genistein in active pulmonary tuberculosis patients during anti-tuberculosis chemotherapy with isoniazid-rifampin-pyrazinamide-ethambutol (HRZE). Indian J. Microbiol. 62, 374–383. 10.1007/s12088-022-01003-2 35974910 PMC9375812

[B80] Montaner J. Ramiro L. Simats A. Tiedt S. Makris K. Jickling G. C. (2020). Multilevel omics for the discovery of biomarkers and therapeutic targets for stroke. Nat. Rev. Neurol. 165 (16), 247–264. 10.1038/s41582-020-0350-6 32322099

[B82] Nieto L. M. R. Mehaffy C. Islam M. N. Fitzgerald B. Belisle J. Prenni J. (2018). Biochemical characterization of isoniazid-resistant *Mycobacterium tuberculosis*: Can the analysis of clonal strains reveal novel targetable pathways? Mol. Cell. Proteomics 17, 1685–1701. 10.1074/MCP.RA118.000821 29844232 PMC6126390

[B83] Opperman M. Loots D. T. van Reenen M. Ronacher K. Walzl G. du Preez I. (2021). Chronological metabolic response to intensive phase TB therapy in patients with cured and failed treatment outcomes. ACS Infect. Dis. 7, 1859–1869. 10.1021/acsinfecdis.1c00162 34043334

[B84] Pal R. Hameed S. Kumar P. Singh S. Fatima Z. (2017). Comparative lipidomics of drug sensitive and resistant *Mycobacterium tuberculosis* reveals altered lipid imprints. 3 Biotech. 7, 325. 10.1007/s13205-017-0972-6 PMC5602786 28955622

[B85] Parihar R. Shukla R. Baishya B. Kalita J. Haldar R. Misra U. K. (2022). NMR based CSF metabolomics in tuberculous meningitis: Correlation with clinical and MRI findings. Metab. Brain Dis. 37, 773–785. 10.1007/S11011-021-00860-Y 35029797

[B86] Pitaloka D. A. E. Syamsunarno M. R. A. A. Abdulah R. Chaidir L. (2022). Omics biomarkers for monitoring tuberculosis treatment: A mini-review of recent insights and future approaches. Infect. Drug Resist. 15, 2703–2711. 10.2147/IDR.S366580 35664683 PMC9160605

[B87] Reedoy K. S. Loots D. T. Beukes D. Reenen M. Pillay B. Pillay M. (2020). *Mycobacterium tuberculosis* curli pili (MTP) is associated with significant host metabolic pathways in an A549 epithelial cell infection model and contributes to the pathogenicity of *Mycobacterium tuberculosis* . Metabolomics 16, 116. 10.1007/s11306-020-01736-5 33084984

[B88] Rêgo A. M. Alves da Silva D. Ferreira N. V. de Pina L. C. Evaristo J. A. M. Caprini Evaristo G. P. (2021). Metabolic profiles of multidrug resistant and extensively drug resistant *Mycobacterium tuberculosis* unveiled by metabolomics. Tuberculosis 126, 102043. 10.1016/J.TUBE.2020.102043 33370646

[B89] Sakallioglu I. T. Barletta R. G. Dussault P. H. Powers R. (2021). Deciphering the mechanism of action of antitubercular compounds with metabolomics. Comput. Struct. Biotechnol. J. 19, 4284–4299. 10.1016/J.CSBJ.2021.07.034 34429848 PMC8358470

[B90] Sethi S. Brietzke E. (2015). Omics-based biomarkers: Application of metabolomics in neuropsychiatric disorders. Int. J. Neuropsychopharmacol. 19, pyv096–13. 10.1093/ijnp/pyv096 26453695 PMC4815467

[B91] Shivakoti R. Newman J. W. Hanna L. E. Queiroz A. T. L. Borkowski K. Gupte A. N. (2022). Host lipidome and tuberculosis treatment failure. Eur. Respir. J. 59, 2004532. 10.1183/13993003.04532-2020 34375300 PMC9625841

[B92] Sun L. Li J. Ren N. Qi H. Dong F. Xiao J. (2016). Utility of novel plasma metabolic markers in the diagnosis of pediatric tuberculosis: A classification and regression tree analysis approach. J. Proteome Res. 15, 3118–3125. 10.1021/acs.jproteome.6b00228 27451809

[B93] Sun L. Zhang L. Wang T. Jiao W. Li Q. Yin Q. (2019). Mutations of *Mycobacterium tuberculosis* induced by anti-tuberculosis treatment result in metabolism changes and elevation of ethambutol resistance. Infect. Genet. Evol. 72, 151–158. 10.1016/J.MEEGID.2018.09.027 30292007

[B94] Tounta V. Liu Y. Cheyne A. Larrouy-Maumus G. (2021). Metabolomics in infectious diseases and drug discovery. Mol. Omi. 17, 376–393. 10.1039/D1MO00017A PMC8202295 34125125

[B95] Triba M. N. Le Moyec L. Amathieu R. Goossens C. Bouchemal N. Nahon P. (2015). PLS/OPLS models in metabolomics: The impact of permutation of dataset rows on the K-fold cross-validation quality parameters. Mol. Biosyst. 11, 13–19. 10.1039/C4MB00414K 25382277

[B96] Tuyiringire N. Tusubira D. Munyampundu J.-P. Tolo C. U. Muvunyi C. M. Ogwang P. E. (2018). Application of metabolomics to drug discovery and understanding the mechanisms of action of medicinal plants with anti‐tuberculosis activity. Clin. Transl. Med. 7, e29. 10.1186/s40169-018-0208-3 PMC6165828 30270413

[B97] Vabalas A. Gowen E. Poliakoff E. Casson A. J. (2019). Machine learning algorithm validation with a limited sample size. PLoS One 14, e0224365. 10.1371/journal.pone.0224365 31697686 PMC6837442

[B98] van der Ploeg T. Austin P. C. Steyerberg E. W. (2014). Modern modelling techniques are data hungry: A simulation study for predicting dichotomous endpoints. BMC Med. Res. Methodol. 14, 137. 10.1186/1471-2288-14-137 25532820 PMC4289553

[B99] van Laarhoven A. Dian S. Aguirre-Gamboa R. Avila-Pacheco J. Ricaño-Ponce I. Ruesen C. (2018). Cerebral tryptophan metabolism and outcome of tuberculous meningitis: An observational cohort study. Lancet Infect. Dis. 18, 526–535. 10.1016/S1473-3099(18)30053-7 29395996

[B100] Vrieling F. Alisjahbana B. Sahiratmadja E. van Crevel R. Harms A. C. Hankemeier T. (2019). Plasma metabolomics in tuberculosis patients with and without concurrent type 2 diabetes at diagnosis and during antibiotic treatment. Sci. Rep. 9, 18669. 10.1038/s41598-019-54983-5 31822686 PMC6904442

[B101] Vrieling F. Ronacher K. Kleynhans L. van den Akker E. Walzl G. Ottenhoff T. H. M. (2018). Patients with concurrent tuberculosis and diabetes have a pro-atherogenic plasma lipid profile. EBioMedicine 32, 192–200. 10.1016/j.ebiom.2018.05.011 29779698 PMC6020709

[B102] Wang C. Peng J. Kuang Y. Zhang J. Dai L. (2017). Metabolomic analysis based on 1H-nuclear magnetic resonance spectroscopy metabolic profiles in tuberculous, malignant and transudative pleural effusion. Mol. Med. Rep. 16, 1147–1156. 10.3892/mmr.2017.6758 28627685 PMC5562006

[B103] Wang S. Yang L. Hu H. Lv L. Ji Z. Zhao Y. (2022). Characteristic gut microbiota and metabolic changes in patients with pulmonary tuberculosis. Microb. Biotechnol. 15, 262–275. 10.1111/1751-7915.13761 33599402 PMC8719804

[B104] Weiner J. Domaszewska T. Donkor S. Kaufmann S. H. E. Hill P. C. Sutherland J. S. (2020). Changes in transcript, metabolite, and antibody reactivity during the early protective immune response in humans to *Mycobacterium tuberculosis* infection. Clin. Infect. Dis. 71, 30–40. 10.1093/CID/CIZ785 31412355 PMC7312225

[B105] Weiner J. Maertzdorf J. Sutherland J. S. Duffy F. J. Thompson E. Suliman S. (2018). Metabolite changes in blood predict the onset of tuberculosis. Nat. Commun. 9, 5208. 10.1038/s41467-018-07635-7 30523338 PMC6283869

[B106] Weiner J. Parida S. K. Maertzdorf J. Black G. F. Repsilber D. Telaar A. (2012). Biomarkers of inflammation, immunosuppression and stress with active disease are revealed by metabolomic profiling of tuberculosis patients. PLoS One 7, e40221. 10.1371/JOURNAL.PONE.0040221 22844400 PMC3402490

[B107] Westerhuis J. A. Hoefsloot H. C. J. Smit S. Vis D. J. Smilde A. K. van Velzen E. J. J. (2008). Assessment of PLSDA cross validation. Metabolomics 4, 81–89. 10.1007/s11306-007-0099-6

[B108] WHO (2022). Global tuberculosis report 2022. Geneva Available at: https://www.who.int/publications/i/item/9789240061729 (Accessed November 1, 2022).

[B109] WHO (2021). WHO announces updated definitions of extensively drug-resistant tuberculosis. Available at: https://www.who.int/news/item/27-01-2021-who-announces-updated-definitions-of-extensively-drug-resistant-tuberculosis (Accessed November 11, 2022).

[B110] Wood P. L. Tippireddy S. Feriante J. (2018). Plasma lipidomics of tuberculosis patients: Altered phosphatidylcholine remodeling. Futur. Sci. OA 4, FSO255. 10.4155/fsoa-2017-0011 PMC5729594 29255627

[B111] Xu Y., P. Borah K. (2022). *Mycobacterium tuberculosis* carbon and nitrogen metabolic fluxes. Biosci. Rep. 42, 20211215. 10.1042/BSR20211215

[B113] Yi W.-J. Han Y.-S. Wei L.-L. Shi L.-Y. Huang H. Jiang T.-T. (2019). l-Histidine, arachidonic acid, biliverdin, and l-cysteine-glutathione disulfide as potential biomarkers for cured pulmonary tuberculosis. Biomed. Pharmacother. 116, 108980. 10.1016/j.biopha.2019.108980 31125821

[B114] Yu Y. Liang H.-F. Chen J. Li Z.-B. Han Y.-S. Chen J.-X. (2021). Postpartum depression: Current status and possible identification using biomarkers. Front. Psychiatry 12, 620371. 10.3389/fpsyt.2021.620371 34211407 PMC8240635

[B115] Yuan T. Werman J. M. Sampson N. S. (2021). The pursuit of mechanism of action: Uncovering drug complexity in TB drug discovery. RSC Chem. Biol. 2, 423–440. 10.1039/D0CB00226G 33928253 PMC8081351

[B116] Zhang P. Zhang W. Lang Y. Qu Y. Chen J. Cui L. (2019). 1H nuclear magnetic resonance-based metabolic profiling of cerebrospinal fluid to identify metabolic features and markers for tuberculosis meningitis. Infect. Genet. Evol. 68, 253–264. 10.1016/J.MEEGID.2019.01.003 30615950

[B117] Zhang P. Zhang W. Lang Y. Qu Y. Chu F. Chen J. (2018). Mass spectrometry-based metabolomics for tuberculosis meningitis. Clin. Chim. Acta 483, 57–63. 10.1016/j.cca.2018.04.022 29678632

[B118] Zhao H. Si Z. H. Li M. H. Jiang L. Fu Y. H. Xing Y. X. (2017). Pyrazinamide-induced hepatotoxicity and gender differences in rats as revealed by a 1H NMR based metabolomics approach. Toxicol. Res. (Camb). 6, 17–29. 10.1039/C6TX00245E 30090474 PMC6062402

[B119] Zhou A. Ni J. Xu Z. Wang Y. Zhang H. Wu W. (2015). Metabolomics specificity of tuberculosis plasma revealed by 1H NMR spectroscopy. Tuberculosis 95, 294–302. 10.1016/J.TUBE.2015.02.038 25736521 PMC4428961

